# How Surface Vacancies Alter Electrocatalytic CO_2_ Reduction Pathways on Cu(111): Insights from Embedded Correlated Wavefunction Theory

**DOI:** 10.1021/acs.jpcc.6c03598

**Published:** 2026-10-14

**Authors:** Vidushi Sharma, John Mark P. Martirez, Emily A. Carter

**Affiliations:** † Applied Materials and Sustainability Sciences, 6740 Princeton Plasma Physics Laboratory, Princeton, New Jersey 08540-6655, United States; ‡ Department of Mechanical and Aerospace Engineering, Princeton University, Princeton, New Jersey 08544-5263, United States; § Andlinger Center for Energy and the Environment and Program in Applied and Computational Mathematics, Princeton University, Princeton, New Jersey 08544-5263, United States

## Abstract

Point defects modify the local coordination environment of crystalline surfaces, creating distinct morphological features accessible to reaction intermediates during catalysis. Here we investigate how surface vacancies on a Cu(111) electrode influence the thermodynamics of primary electrochemical CO_2_ reduction intermediates and further alter the kinetics of CO hydrogenation. Using a density functional embedding framework, we combine a higher-accuracy embedded correlated wavefunction theory (ECW) treatment of the active region with a density functional theory (DFT) description of the extended electrode. ECW predicts a vacancy-induced relative stabilization of primary intermediates, especially CO and CHO (formyl), next to the defect, while COH (hydroxymethylidyne) remains largely unaffected relative to pristine (defect-free) Cu(111). Based on earlier ECW findings favoring proton-coupled electron transfer (PCET) mechanisms, here we study competing PCET electrochemical CO reduction pathways to CHO and COH, first at the DFT level, followed by ECW refinement. While DFT predicts both pathways to be kinetically accessible with a slight preference for COH formation, ECW systematically increases activation barriers while maintaining a lower barrier along the COH pathway. At experimental onset potentials for hydrocarbon production on Cu(111) (−0.9 V vs RHE), ECW predicts exergonic CHO formation but with a substantial activation barrier, whereas COH formation remains endergonic but kinetically much more accessible. *Our results indicate that, under typical operating conditions, defect-engineered Cu(111) surfaces with vacancies will exhibit kinetic selectivity toward the COH pathway – like Cu(100) but in contrast to pristine Cu(111), where the latter has an equal propensity for both pathways at the experimental onset potential of −0.9 V vs RHE.*

## Graphical abstract

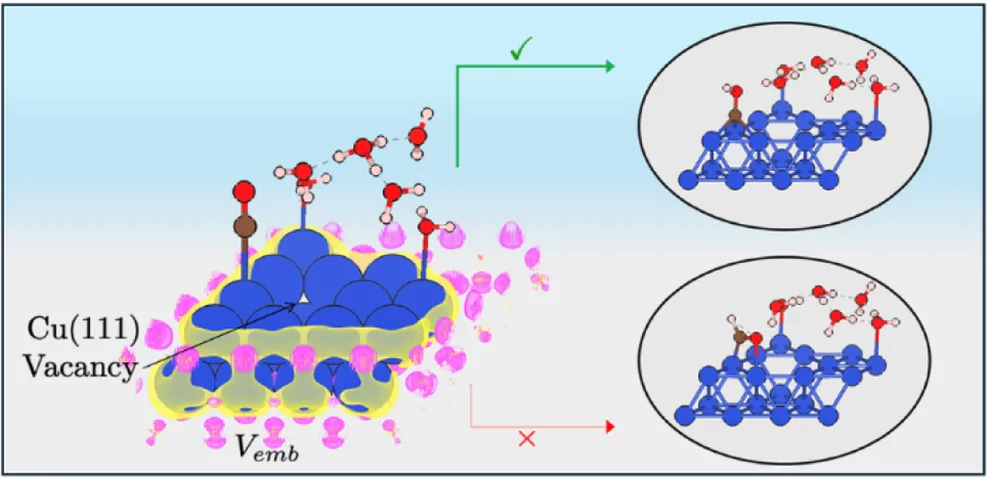

## 1 Introduction

Rapid advances in the electrochemical conversion of carbon dioxide^1–8^ into chemical feedstocks offer a compelling pathway to turn waste emissions into useful products and close the carbon cycle. For decades, most research efforts have focused on identifying ways to improve catalyst efficiency and selectivity toward desirable product formation, such as higher C_2+_ hydrocarbons and alcohols.

A myriad of experimental and theoretical studies have examined the facet-dependent activity^9–13^ of electrocatalysts, further informing the design of electrocatalysts for electrochemical CO_2_ reduction (eCO_2_R). Yet *the dependence of the CO*_2_ *reduction paths on the presence of surface point defects is not fully understood*. The occurrence of point defects alters the local coordination environment, which can influence significantly the binding of eCO_2_R intermediates and their related reaction kinetics. Soft metals such as copper (Cu) readily form defects, and their oxide derivatives are known to sustain vacancies due to missing oxygen atoms in the (sub)­surface layers. Indeed, oxide-derived Cu (Cu oxides thereafter reduced to Cu) was discovered quite some time ago to exhibit enhanced eCO_2_R reactivity.^14,15^ Numerous defect-engineering studies in metal oxides^11,16–21^ have explored a broad range of dopants, vacancies, defects, interfaces, and atomic substitutions with the goal of improving their electrocatalytic activity or selectivity. However, a fundamental mechanistic understanding of how each of these perturbations to a pristine (defect-free) electrode affects the overall reaction mechanism and outcome remains incomplete. In the present study, we isolate the role of surface vacancies on Cu(111) to deconvolute their effect from other structural perturbations such as surface reconstruction and oxygen incorporation.

Using in situ surface-enhanced Raman spectroscopy in conjunction with density functional theory (DFT), Zhan et al.^22^ recently showed that undercoordinated lattice sites and compression-induced distortions in Cu enhance eCO_2_R to ethylene and ethanol. Much of their work featured DFT- generalized-gradient approximation (GGA)^23^-based vibrational frequency analysis to identify and distinguish the vibrational modes of various carbon monoxide (CO) reduction intermediates as seen in Raman spectra. They also computed DFT binding energies of CO adsorbed atop a Cu atom on various defective and oxide-derived Cu models and found CO to be more strongly bound in the presence of any imperfection as compared to pristine Cu morphology.

It is well known that DFT-GGA^23^ fails to produce the correct binding energy and preferred adsorption site of CO on Cu,^24,25^ owing to electron self-interaction/delocalization error in the approximate exchange-correlation (XC) functional. Sharifzadeh et al.^26^ demonstrated that this deficiency is remedied by embedded correlated wavefunction theory (ECW), which, by virtue of including exact exchange and dynamical correlation in the active region, corrects DFT-GGA’s excess *π*-backbonding from the Cu surface to the adsorbed CO – the origin of the errors in binding energy and site preference. In our current study described below, we utilize DFT prescreening followed by an ECW refinement of the primary adsorbate-metal interactions critical to eCO_2_R to avoid such artifacts.

In an earlier related study, Scholten et al.^27^ identified surface roughness or defects – introduced via electrochemical etching or oxygen-plasma treatment – as critical for hydrocarbon production via electrolysis on single-crystalline Cu(100) and Cu(111) facets. They also reported a correlation between the CO binding strength and electrocatalytic selectivity toward hydrocarbons over H_2_, based on CO desorption energies derived from temperature-programmed desorption data across their sample surfaces. More notably, their surface characterization experiments showed that clean and atomically ordered flat copper surfaces, both Cu(100) and Cu(111), instead favor the competing hydrogen evolution reaction. These observations are in line with prior reports of strong CO-binding sites on CuO_*x*_ electrodes generated via controlled oxygen-plasma treatment,^28^ which undergo subsequent transformation to metallic Cu under eCO_2_R reaction conditions.

Motivated by recent experimental evidence that surface defects on low-index Cu facets can enhance eCO_2_R,^22,27,29^ here we theoretically investigate the specific effect of Cu surface vacancies on electrochemical CO reduction (eCOR) pathways, since the latter are often thought to be rate-determining in eCO_2_R. We first evaluate adsorption-site preferences of the eCO_2_ primary reduction intermediates, namely, CO, formyl (CHO, with a carbonyl group), and hydroxymethylidyne (COH, with a hydroxyl group), at candidate sites on Cu(111) in the presence of a nearby surface vacancy. This enables identification of the “active” sites for *CO reduction pathways to form *CHO and *COH, where the asterisk (*) next to the species indicates it is adsorbed. We then simulate proton-coupled electron transfer (PCET) kinetics, shown previously to be the most favorable paths on pristine Cu surfaces.^30,31^ We determine the minimum energy paths (MEPs), reaction free-energies, and free-energy barriers for the first step in CO reduction, namely hydrogenation of *CO. Each step in our modeling of eCO_2_R is first carried out using planewave (PW) DFT-GGA and then the electronic structure is locally refined with a more accurate, higher-level correlated wavefunction (CW) theory in the presence of an embedding potential that represents the extended metal surface. The embedding potential is derived from density-functional embedding theory (DFET; *vide infra*). We then utilize the Chan−Nørskov capacitor model^32,33^ to convert constant-charge energetics to constant-potential ones, by computing surface work functions and charge transfer along the reaction paths, as is necessary to derive potential-dependent reaction free energies and free-energy barriers for the electroreduction of *CO to *CHO and *COH. We find that surface vacancies qualitatively alter the kinetics of these two pathways as compared to the pristine surface, thereby strongly modifying the initial branching of the first *CO PCET step.

## 2 Computational Methodology

### 2.1 Model Setup for Periodic PW-DFT

We performed an analysis for the preferred adsorbed configurations of CO, COH, and CHO on a Cu(111) surface with a nearby surface vacancy using a one-sided [1S] periodic slab model in vacuum, as was done in our previous studies; see Supporting Information (SI) Section S1, Figure S1 for all adsorption sites.^30,31,34,35^ Since all adsorbates are charge-neutral, this [1S] model is adequate for an efficient preliminary screening. We later switch to a two-sided [2S] slab model to achieve physically correct charge localization under implicit solvation (*vide infra*).^36^ The [1S] model consists of a five-layer (4 × 4)-supercell slab of Cu(111) with 15 Å of vacuum separating the periodic images along the surface normal direction. A vacancy in the surface layer yields a 79-atom Cu(111) periodic slab. Geometric optimizations were performed using dipole-field corrections (applied to both energy and potential) in vacuum^37^ to avoid unphysical interactions between periodic images across the vacuum. We performed all PW-DFT calculations with VASP 6.4.3.^38,39^ We employed the projector augmented-wave (PAW) method within PW-DFT, utilizing all-electron PAW screened ion potentials within the frozen-core approximation^40^ to self-consistently solve for the valence electrons in Cu­(11*e* ^–^), O­(6*e* ^–^), C­(4*e* ^–^), and H­(1*e* ^–^), all taken from the PAW_64 data set.^41^ We chose a PW kinetic energy cutoff of 660 eV, an “accurate” precision setting for the FFT grid, and a Γ-point-shifted Monkhorst–Pack^42^ grid of 4 × 4 × 1 to sample *k*-space. We use the Methfessel−Paxton scheme^43^ for Brillouin zone integration, with a smearing width of 0.09 eV. These parameter choices are sufficient to converge the total energy to within 1 meV/atom. We used the Perdew–Burke–Ernzerhof GGA XC functional (GGA-PBE)^23^ along with D3­(BJ) van der Waals dispersion corrections with Becke−Johnson damping.^44–48^ We performed structural optimizations using a conjugate-gradient algorithm until the maximum residual force on a given atom was below 0.02 eV/Å, with all atoms allowed to relax unless otherwise noted. In the [1S] model, we relaxed the adsorbates and the top two layers of Cu(111) slab, while freezing the bottom three layers to represent the bulk boundary condition, to be consistent with the number of layers relaxed in the [2S] model (*vide infra*).^35,36^

### 2.2 Implicit (Continuum) Plus Explicit Water Solvation Models and PCET Pathway Optimization

Starting from the optimized geometries in vacuum (*vide supra*), we replaced the vacuum with continuum water represented by a bulk-water dielectric constant of *ε_b_* = 78.4, in the periodic PW-DFT slab calculations. We use the VASPsol^49–51^ interface, which solves the (linearized) Poisson–Boltzmann equation to model implicit solvation in periodic PW-DFT calculations. We set the surface tension parameter for the solvation cavity in the continuum model^51^ *τ* to zero to eliminate the cavitation term, which by default is parameterized to reproduce molecular solvation energetics.^52^ In so doing, we retain only the electrostatic/electrolyte screening response of the continuum solvent relevant for planar metal-solution interfaces. Setting *τ* = 0 avoids any numerical instability (noise) and facilitates convergence of the continuum bulk electrostatic potential for metal-slab/solution interfaces without affecting the energetics, see SI Section S2A.

This continuum dielectric representation of water as implemented in VASPsol^49,51,53^ is particularly useful for describing the reduction of *CO to *CHO/*COH via PCET, where an implicitly solvated Eigen cation [(H_3_O^+^)­(H_2_O)_3_ or H_9_O_4_^+^] acts as the proton donor. It was previously established that *CO reduction on Cu(111) and Cu(100) is more likely to proceed via a PCET process – potential-dependent by nature – instead of a direct surface hydrogen-atom transfer mechanism, especially with increasingly negative applied potentials.^30,31^ Consequently, we construct a hybrid explicit-implicit model to account for both short-range (explicit) and long-range (implicit, continuum) solvent response during PCET reduction of *CO as facilitated by an Eigen cation in the electric double layer. We form, as done previously,^30,31,54,55^ the Eigen cation by placing a neutral (H_3_O)­(H_2_O)_3_ species above the surface (*vide infra*), which autoionizes to form the cation in polarizable continuum water, with its excess electron absorbed by the metal surface. Because the Eigen cation H_9_O_4_^+^ is the explicit proton donor, the resulting barriers describe a hydronium-mediated PCET pathway that is obviously most directly applicable to acidic solutions or locally hydronium-rich interfacial conditions (the latter favored by the negatively charged electrode). At neutral to alkaline pHs, H_2_O has been proposed as a possible proton donor (see, e.g., refs 56 and 57) albeit with much larger PCET barriers than from hydronium due to the required heterolytic O–H bond breaking in water^57^ that may render such a pathway irrelevant (e.g., the 0.7 eV higher PCET barrier for water rather than hydronium as the proton source reported in ref 57 translates to a ∼10^12^ reduction in rate at room temperature for water-derived PCET). The present model does not describe this water-mediated pathway but only focuses on the more likely hydronium-derived PCET pathway, which still may dominate in neutral to alkaline solutions despite being present in very low concentrations because the rate depends only linearly on concentration while exponentially on the barrier.

To ensure a physically consistent treatment of the PCET water arrangement across structures ranging from *CO to *CHO/*COH, we introduce in the model two additional spectator waters adsorbed directly on the surface (*vide infra*), in order to prevent the water molecules constituting the Eigen cation from adsorbing onto the surface over the course of the reaction; were Eigen-cation-derived water adsorption to occur, it would introduce a spurious energy contribution not relevant to CO reduction energetics. The reaction paths here ensure that the general structure of the solvent is maintained along the path via local relaxation, except when directly involved in proton transfer, and does not include spurious water adsorption contributions. Ideally, one would explicitly sample liquid water configurations from molecular dynamics (MD) simulations of an electrode–electrolyte interface model undergoing CO reduction.^58–60^ Such sampling to obtain solvent-ensemble-averaged free energies is beyond the scope of the present ECW workflow for heterogeneous catalysis. However, as we recently integrated ECW with MD sampling for aqueous-phase reactions,^61–67^ we hope to include such sampling in future interfacial studies. For now, maintaining an intact solvated Eigen cation helps ensure that the PCET energetic changes obtained here are purely due to the action of the proton transfer, and not from water adsorption or rearrangement (the latter being unlikely for the Eigen cation reactant due to its strong hydrogen bonding, while after proton transfer, the resulting water cluster may rearrange on longer time scales not relevant to the PCET event).

To eliminate any lateral interactions involving the PCET-related species, we employ larger (5 × 5) periodic slabs of Cu(111) with varying thickness (*vide infra*, using an unshifted 3 × 3 × 1 k-mesh) for determining the PCET reaction pathways. The atomic coordinates for each model of reactants and products are provided as SI files. At first, we consider a hybrid-solvation model with the surface and solution species located only on one side of the slab ([1S]-sol). However, this results in a spurious charge localization on the other side of the periodic slab, i.e., the side without adsorbates (see SI Section S2). Notably, this effect is not immediately obvious from the electrostatic potential but rather requires an inspection of the charge densities. To address this issue, we then utilize a more apt [2S]-sol model in which the adsorbates are symmetrized (via shift and translation) on the opposite side of the slab. This construction ensures a (quantifiable) physical surface charge localization on both sides of the slab in contact with the implicit solvent; further details are provided in SI Section S2 and Figures S2–S4. The [2S]-sol model requires an expansion of the simulation cell along the surface normal direction, together with the addition of Cu bulk layers, yielding a six-layer (5 × 5) periodic slab. In PW-DFT simulations using the [2S]-sol model, the two central Cu layers are fixed in their equilibrium bulk positions to set a bulk-like boundary condition for the surface layers, while the two outer Cu layers on each side and the adsorbates plus the Eigen cation/waters in solution are allowed to relax (fixing the adsorbed spectator waters at their optimized initial state coordinates and the lateral, hereafter denoted as (*x*, *y*), coordinates of C in *CO, constraining it to not move laterally and remain atop, *vide infra*); this setup is denoted as *2 | 2 | 2*, see SI Sections S3 (Figure S5) and S4.

At negative potentials, solvated cations accumulate near the electrode and together with their image charges in the metal, generate an interfacial electric field. Cation identity, concentration, hydration structure, interfacial position, and applied potential determine the magnitude of the interfacial field and its influence on CO and CO_2_ reduction kinetics.^68^ Electrochemical mass spectrometry and HPLC experiments revealed that large alkali cations can enhance C_2_H_4_ formation at Cu electrodes at relatively low overpotentials, particularly on Cu(100). Because in our symmetric [2S]-sol model, the equal and oppositely oriented local dipoles at the two interfaces cancel, modeling cation-induced electric fields with a uniform field applied along the surface-normal of the cell would act with opposite signs at each individual interface, an unphysical condition. A physical representation of cation-induced field effects would therefore require either an asymmetric electrochemical-interface model that would yield unphysical charge distributions in the slab or, more appropriately, a properly charged symmetric double-layer construction. Given the complexity of the latter, we leave the investigation of cation effects on defect-containing Cu surfaces for future work.

The PCET MEPs computed via the climbing-image–nudged elastic band (CI-NEB) method^69,70^ are first determined in vacuum ([1S]) for computational efficiency; the converged structures then become initial guesses for CI-NEB in [2S]-sol (and [1S]-sol for comparison, see SI Section S4, Figures S6–S8). In all cases, the initial states (ISs; reactants) and final states (FSs; products) are constrained to adopt the structures associated with the ECW-predicted lowest energy adsites (*vide infra*). As noted above, the solvent coordinates for H_9_O_4_^+^ at the IS, four H_2_Os at the FS, and the related solvent species in the intermediate states, are all allowed to relax during optimization of the constant-electron DFT MEP. However, water orientations (e.g., H-down, O-down, and intermediate) are known to depend on the electrode potential,^71,72^ which our constant-electron-based structures do not necessarily sample. To properly do so would require dynamical sampling of the metal-solution interface under constant potential (grand canonical ensemble) conditions, and subsequently repeating the extensive computations done here for multiple solvent configurations, which is prohibitively expensive. That said, neglecting this effect as we do is likely a reasonable approximation, since the always-negative electrode potentials relevant to CO reduction will have a strongly preferred orientation for water and water-containing species at, e.g., −1 V vs RHE.

We include vibrational energy contributions in the Gibbs free energy from all adsorbates, solution species (Eigen cation or waters), and Cu atoms belonging to the outermost layer of the Cu(111) slab. In [2S]-sol models, we account for the vibrations from each set of symmetrized adsorbates, solution species, and their adjacent Cu surface layer on both sides of the slab. To achieve this, VASP diagonalizes partial numerical Hessians constructed from finite differences of atomic displacements of ± 0.01 Å along each Cartesian coordinate away from each critical point, for which VASP computes the resulting atomic forces. From the vibrational frequencies thus computed, we also verify that the ISs and FSs chosen for the MEP correspond to minima, and that the transition state (TS) is a first-order saddle point.

### 2.3 DFET/ECW Calculations

To refine the electronic structure description of the eCO_2_R active sites, we perform CW calculations in the presence of an embedding potential (*V_emb_*) derived using DFET.^73–76^ We utilize ECW theory instead of emerging periodic CW methods here because the former is much more computationally efficient and the latter do not yet include multireference methods required for studying reactions on metal surfaces. Our DFET framework is set up in VASP (VASPEmbedding)^77^ and the subsequent ECW calculations are performed with Molpro v. 2024.1 ^78,79^ using Gaussian-type orbital (GTO) basis sets. We use the *EmbeddingIntegralGenerator* code (available on GitHub)^80^ to convert the *V_emb_* from a real-space grid to a GTO basis set representation for use in quantum chemistry codes. The resulting potential integral file is input to Molpro via the MATROP routine.

To enable ECW calculations, we must first obtain a representation of the interactions between a small fragment consisting of the active region (a cluster of atoms involved in the phenomenon of interest that requires a refined CW description) and its extended environment. The periodic slab model in vacuum (*vide supra*) hence was divided into a region of interest, the *cluster*, and the remainder was classified as its *environment*. Following this real-space partitioning, we optimize a *V_emb_* that mimics the effect of the environment on a given cluster and vice versa; this DFET *V_emb_* is unique to the cluster and environment and is exact within the DFT-XC approximation utilized. Crucially, *V_emb_* is generated for a self-consistent set of electron densities of the cluster (*ρ_cluster_*) and its environment (*ρ_env_*), each subject to the self-consistently optimized *V_emb_*. At convergence of *V_emb_*, the sum of these two densities reproduces the total electron density of the full system (*ρ_tot_*); see the second line of eq 1 where one sees this density difference defines the gradient for optimizing *V_emb_*. The unique *V_emb_* is determined by maximizing an extended Wu−Yang functional^81^ (minimizing −*W*[*V_emb_*] using an LBFGS optimizer)^82^ defined as,

1 $$\begin{array}{ll} W\left[V_{emb}\right] & =E_{cluster}\left[\rho_{cluster};V_{emb}\right]+E_{env}\left[\rho_{env};V_{emb}\right]-\int \rho_{tot}\left(r\right)\cdot V_{emb}\left(r\right)dr,\\ \frac{\delta W\left[V_{emb}\right]}{\delta V_{emb}\left(r\right)} & =\rho_{cluster}\left(r\right)+\rho_{env}\left(r\right)-\rho_{tot}\left(r\right) \end{array}$$

where *E_cluster_* and *E_env_* are the self-consistent PW-DFT energies of the cluster and its environment in the presence of *V_emb_*, respectively. The converged *V_emb_* is written on a uniform real-space grid. Here, *V_emb_* is computed for metal clusters only (i.e., without adsorbates), as our goal is to describe the metal–metal (cluster–environment) interactions^83^ within DFET. This enables the same *V_emb_* to be consistently applied to all structures along a given MEP.

We employed two embedded (emb) clusters, emb-Cu_16_ and emb-Cu_15_, to compare adsites for *CO and *COH/*CHO, respectively, as shown in Figure 1. These cluster sizes and shapes ensure that the total number of electrons of the cluster with the adsorbates is even (avoiding spurious spin polarization in the ECW calculations), the metal’s density distribution is multilayer (a physically appropriate distribution), and that all direct molecule–surface interactions (with positions determined by the DFT MEPs) are contained within the cluster and are thus fully quantum mechanically described.^83^ Following the same principle for selecting cluster sizes and shapes, we used an emb-Cu_19_ cluster for structures along the MEPs of the two PCET reactions, namely, *CO → *COH and *CO → *CHO. All clusters were carved out of a four-layer (5 × 5) Cu slab to minimize interactions between periodic images of the adsorbates as well as to derive a spatially well-separated *V_emb_* along the *xy*-plane; see SI Section S5 and Figure S9 for *V_emb_*.

**Figure 1:**
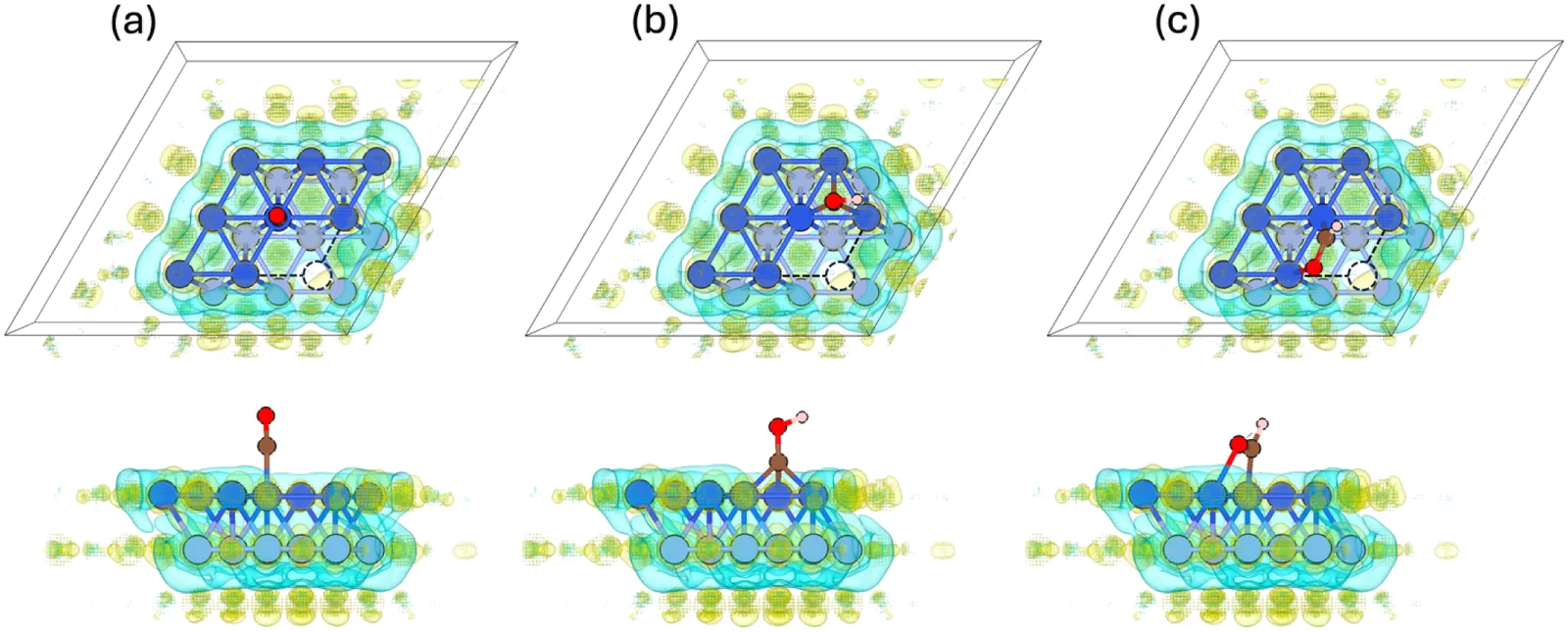
ECW-preferred geometries of eCO_2_R intermediates: (a) *CO (atop), (b) *COH (hollow-*3c*), (c) *CHO (vacancy-adjacent bidentate-*v*) shown on Cu(111) embedded clusters with a vacancy indicated by the dashed circle. Top (bottom) row shows the top-down (side) view of the atomic structures, where dark and light-blue filled spheres indicate surface and subsurface Cu, respectively, and brown, red, and white filled spheres represent C, O, and H, respectively. Isosurface plots (yellow, +1 V; cyan, −1 V) represent the *V_emb_* generated for (a) Cu_16_ (environ: 83 Cu) and (b) and (c) Cu_15_ (environ: 84 Cu) embedded clusters.

Following DFET *V_emb_* optimization, we then add *V_emb_* expressed in the chosen atomic orbital basis to the cluster’s one-electron Hamiltonian matrix to include the cluster-environment interactions in the CW calculations.^73–76^ The final ECW energy is defined as

2 $$E^{ECW}=E_{slab}^{PW-DFT}+\left(E_{emb-cluster}^{CW}-E_{emb-cluster}^{DFT}\right)$$

where here CW denotes CASPT2 or NEVPT2 (*vide infra*). $E_{slab}^{PW-DFT}$ here is the PW-DFT-PBE energy of the periodic slab removing the D3­(BJ) dispersion corrections to avoid double counting in the embedded region (van der Waals interactions are accounted for *ab initio* by the PT2 there), $E_{emb-cluster}^{CW}$ and $E_{emb-cluster}^{DFT}$ are the CW and DFT-PBE (without D3­(BJ)) energies of the cluster, respectively, evaluated in the presence of *V_emb_* using the GTO bases. Thus, the self-interaction/delocalization errors of semilocal DFT-GGA are regionally corrected for catalytically active sites by ECW theory, while simultaneously describing the static extended (metal) environment at an appropriate DFT level (GGA).

Note that the geometries for the molecules added to the embedded clusters are generated by mapping the PW-DFT-optimized adsorbate/solution species structures onto the emb-Cu clusters using the vacancy as the reference point; see, e.g., SI Figure S1. Although the surface vacancy appears near the edge of the embedded clusters in Figure S1, it should not be interpreted as an edge defect. The vacancy is solved for – its geometry and electron density, and *V_emb_* – in the presence of the rest of the periodic slab at the DFT level. However, the embedded clusters and relative adsorbate positions on them are constructed to have the adsorbates not interacting directly with *V_emb_*. *V_emb_* is intended to reproduce metal–metal bonding interactions between the cluster and its environment but is not aware of direct adsorbate-environment interactions. The vacancy thus ends up closer to *V_emb_*, with metal–metal bonding at the vacancy properly reproduced by *V_emb_* by construction.

We first use a multiconfigurational self-consistent field approach – Complete Active Space Self-Consistent Field (CASSCF) theory,^84^ within the DFET/ECW framework to obtain a good qualitative description of static electron correlation. Our objective with emb-CASSCF is to identify an appropriate active space (AS) of *n* electrons distributed among *m* orbitals, written as (*n,m*), such that the AS includes the relevant metal–adsorbate orbital interactions and static electron correlation effects. Such effects are crucial for accurately describing bond formation and scission; our active spaces are chosen to include orbitals participating in such processes. The optimized emb-CASSCF­(*n,m*) wavefunctions are utilized then as reference wavefunctions for embedded Complete Active Space second-order Perturbation Theory (emb-CASPT2)^85^ and embedded *N*–Electron Valence state second-order Perturbation Theory (emb-NEVPT2)^86^ calculations, to include the dynamic electron correlation effects needed for a quantitative comparison across structures. We employed a 10-electron LANL effective core potential for Cu and its corresponding LANL2DZ basis set,^87^ and the all-electron cc-pVDZ basis sets for C, O, and H.^88–91^ We used the resolution-of-the-identity (RI) approximation^92^ in evaluating the two-electron integrals to speed up the CW calculations and the corresponding RI auxiliary basis sets (MP2FIT in Molpro).^93^

The emb-CASSCF AS for adsite screening are all (12e,12o).^94^ For *CO adsites, the AS consists of the following electron pairs and their correlating virtual orbitals: C–O dative pair 5*σ*,6*σ* (2e,2o), two C–O covalent *π*,*π* ^*^ – (4e,4o), the C lone pair and a correlating virtual orbital CO 4*σ*,7*σ* – (2e,2o), and two Cu-4*s* metal–metal pairs – (4e,4o) – on the Cu_16_ cluster containing the vacancy. For ^*^COH adsites, the AS comprises the following electron pairs and correlating virtual orbitals: three C–Cu *σ*,*σ* ^*^ – (6e,6o), C–O *σ*,*σ* ^*^ – (2e,2o), O–H *σ*,*σ* ^*^ – (2e,2o), and one O *sp* ^3^-lone pair – (2e,2o). For ^*^CHO adsites, the AS consists of the C–H *σ*,*σ* ^*^ – (2e,2o), C–O *σ*,*σ* ^*^ – (2e,2o) and *π*,*π* ^*^ – (2e,2o), C–Cu *σ*,*σ* ^*^ – (2e,2o), an O–Cu dative bond from an O lone pair to Cu – (2e,2o), and one nonbonding O *sp* ^3^-lone pair of electrons and its correlating virtual orbital – (2e,2o).

The PCET model for the ECW-corrected MEP comprises an Eigen cation (H_9_O_4_^+^), two spectator H_2_O molecules, and *CO on emb-Cu_19_ as the IS, and correspondingly four H_2_O molecules (the conjugate base of the Eigen cation), two spectator H_2_O molecules, and either *COH or *CHO on emb-Cu_19_ as the FS, *vide infra*. The neutralizing electron is present on the emb-Cu_19_ cluster just like it is in the Cu slab in PW-DFT; confining the electron to within the atoms in the cluster is physically appropriate, given PCET to *CO involves localized electron transfer from that region of the surface where the adsorbate is present. We chose the AS to describe the evolving interactions and electron transfer between the adsorbates and the Cu surface along the MEP. We selected two AS sizes, (14e,14o) and (12e,12o); the smaller AS was required for emb-NEVPT2 for computational tractability. In constructing the AS, we ensured that orbitals critical to the proton transfer were included, while consistently retaining a pair of correlating orbitals near the Cu Fermi level for all MEP structures.^30,94^ The AS was determined using a combined “merge and creep” strategy.^83^ AS orbitals for the IS and FS were first obtained via merging (i.e., combining the CAS natural orbitals optimized separately for the adsorbate and the embedded Cu cluster), followed by creeping from both directions toward the TS. In creeping, we use the optimized CAS natural orbitals from one structure to initialize the CASSCF for another structure slightly different in geometry. At the TS, two candidates for the AS – each initialized from one direction – were evaluated, and the lower CASSCF-energy solution was selected.

In emb-CASPT2 calculations, we apply an ionization potential–electron affinity (IP-EA) shift^95^ = 0.25 Ha to eliminate intruder states, along with a level shift^96^ = 0.30 Ha to aid in numerical convergence. These values are chosen to match those used in ref 30, thereby enabling a consistent comparison between vacancy-containing and pristine Cu(111) surfaces, without introducing differences arising from different choices of numerical parameters. Since the emb-CASPT2 results can be sensitive to the level-shift choice, we also perform emb-NEVPT2 calculations, which do not rely on such ad hoc numerical parameters (no level shift or IP-EA shift needed) and are free of intruder states by construction^86,97^ but are more computational memory intensive and thus limited in AS size.

## 3 Results and Discussion

### 3.1 Thermodynamic Screening of Adsites for CO_2_ Reduction Intermediates with DFT and ECW Theory

We first examine the effect of a neighboring vacancy on the preferred adsorption configurations of the reaction intermediates (see relative energies given in Table 1). Because adsorption energies for these eCOR intermediates at atomically defined Cu(111) defects such as vacancies have not been measured (in vacuum or aqueous solution), we report only relative adsite energies, as our aim here is simply to identify site and adsorbate geometry preferences for our subsequent kinetics assessments. Although no experimental binding energies for *CHO or *COH on Cu are available, ECW theory computations of CO binding to pristine Cu(111) in vacuum have been validated directly against experiment, agreeing both qualitatively in terms of site preference and quantitatively in terms of binding energy to within 0.05 eV,^26^ lending credence to an ECW-based assessment of adsite preferences in solution. To assess the maximum perturbation, in all cases the adsorbate was placed adjacent to the vacancy (structures depicted in SI Figure S1). The inclusion of zero-point-energy (ZPE) corrections modifies the relative energies by a maximum of 8 meV for *CO, 19 meV for *COH, and 30 meV for *CHO, with no changes in the relative stability for any of them (SI Table S1), so below we simply discuss the internal energy differences.

**Table 1:** Relative Energies (eV) for Adsites of *CO, *COH, and *CHO on Cu(111) with a Surface Vacancy Nearby the Adsorbate, Evaluated Using DFT-PBE-D3­(BJ) and ECW (emb-CASPT2, emb-NEVPT2) Methods in Vacuum^a^,^b^,^c^

		atop	bridge-*2c*	hcp-*2c*	fcc-*2c*	hollow-*3c*	bidentate-*v*	bidentate-*p*
*CO	DFT-PBE-D3(BJ)	0.15	0.01	0.02	0	0.07		
	emb-CASPT2	0	0.19	0.29	0.25	0.29		
	emb-NEVPT2	0	0.29	0.34	0.32	0.56		
	R_*xy*_(C–V_Cu_)	2.57	2.23	1.45	1.47	2.94		
*COH	DFT-PBE-D3(BJ)	–^d^	0.13	0.23	0.20	0		
	emb-CASPT2	–	0.16	0.22	0.30	0		
	emb-NEVPT2	–	0.25	0.30	0.33	0		
	R_*xy*_(C–V_Cu_)	–	2.22	1.44	1.47	2.82		
*CHO	DFT-PBE-D3(BJ)	0.28	0.18	0.03	0.03	0.23	0	0.25
	emb-CASPT2	0.57	0.29	0.19	0.38	0.26	0	0.28
	emb-NEVPT2	0.38	0.21	0.37	0.32	0.27	0	0.20
	R_*xy*_(C–V_Cu_)	2.57	2.21	1.45	1.46	2.94	1.82	4.02

a All energies are relative to the energy of the preferred adsite at each level of theory (set to 0). Analogous DFT-PBE-D3­(BJ) energies including ZPE corrections appear in SI Table S1. “*2c*” and “*3c*” refer to 2-fold and 3-fold coordination, respectively. “*v*” refers to the bidentate adsite next to the vacancy while “*p*” indicates a bidentate adsite farther away from the vacancy, mimicking a pristine-surface site. R*_xy_* (C–V_Cu_) (Å) denotes the lateral distance between C and the vacancy site.

b emb-CASPT2 uses a (12e,12o) AS with IP-EA shift = 0.25 Ha and level shift = 0.30 Ha;^30^ emb-NEVPT2 uses a (12e,12o) AS.

c R_*xy*_(C–V_Cu_) indicates the lateral distance between the adsorbate’s C atom and the vacancy site (the latter defined by the position of the missing Cu atom had it been present in a pristine, adsorbate-free surface).

d Atop–*COH is found to be not a local minimum within PW-DFT and hence, is not included.

As expected, for *CO, PW-DFT predicts all hollow and bridge (fcc-*2c*, hcp-*2c*, hollow-*3c,* and bridge-*2c*) sites to lie very close in energy, spuriously stabilizing them relative to the atop site. This artifact is corrected by a more accurate treatment of the electronic structure via ECW theory, which consistently identifies atop as the most favorable adsite for CO, in agreement with a wide variety of surface-characterization experiments.^98–102^ The atomic structure of the atop configuration is shown in Figure 1a. ECW theory further lifts the degeneracy among other PW-DFT adsites listed in Table 1, estimating the nearest distinct configuration (bridge-*2c*) to be higher in energy than atop by 0.29 eV (0.19 eV) at the emb-NEVPT2 (emb-CASPT2) level. The apparent degeneracy in bridge-*2c*, hcp-*2c*, and fcc-*2c* sites in PW-DFT could be attributed to the 2-fold coordination of *CO with the Cu surface at these sites, but this level of theory fails to resolve more nuanced interactions. In contrast, ECW distinguishes between bridge-*2c* – where *CO remains approximately perpendicular to the surface – and the hcp-*2c* and fcc-*2c* configurations, where CO tilts toward the vacancy by approximately 65° and 66° relative to the plane of the surface, respectively, representing a varying extent of CO-Cu orbital interactions. For *CO, there is no obvious correlation between stability and distance from the vacancy (R*_xy_* (C–V_Cu_) in Table 1).

Turning to *COH, PW-DFT favors the hollow-*3c* site as on pristine Cu(111), largely unperturbed by the presence of the surface vacancy (Figure 1b). This preference is further confirmed by ECW calculations at the emb-CASPT2 and emb-NEVPT2 levels, see Table 1. At hollow-*3c*, *COH bonds with three Cu atoms (C–Cu: ∼1.96 Å, C–O: ∼1.34 Å), adopting a distorted tetrahedral geometry with *sp* ^3^-like hybridization. The “*2c*” sites are all 0.2–0.3 eV higher than the “*3c*” site, due to the ability of *sp* ^3^-hybridized C­(OH) to form three bonds in the hollow and only two in the undercoordinated sites. ^*^COH prefers to be as far from the vacancy as possible, based on the R_*xy*_(C–V_Cu_) trend in Table 1. ^*^COH above the vacancy is not a local minimum and relaxes away from the vacancy to coordinate with the nearest surface Cu atoms at bridge-*2c*.

Because of its carbon-centered-radical character, ^*^CHO preferentially adsorbs on vacancy-containing Cu(111) in an atop-bidentate configuration through both the C and O atoms, making respectively covalent and dative bonds to Cu atoms, as illustrated in Figure 1c. Here, the trend is the opposite from ^*^COH with respect to binding near or far from the vacancy, with ^*^CHO preferring to bind near the vacancy (see R_*xy*_(C–V_Cu_)­s in Table 1). The higher degree of Cu coordinative unsaturation at the vacancy favors binding of unsaturated radicals like CHO. More specifically, of the two possible bidentate motifs – one adjacent to the vacancy (bidentate-*v*) and one farther away resembling the pristine surface (bidentate-*p*) – the bidentate-*v* is found to be more stable by 0.25 eV within PW-DFT; at the emb-NEVPT2 (emb-CASPT2) level, it is favored by 0.20 eV (0.28 eV), indicating stronger binding of ^*^CHO at bidentate-*v*, see Table 1. That said, PW-DFT finds hcp-2*c* and fcc-2*c* essentially as stable as bidentate-*v*, in contrast to ECW theory, which finds bidentate-*v* as the definitive, lowest-energy configuration. We shall see that the MEP for *CO → *CHO proceeds via the bridge site, which constitutes the nearest accessible (and next lowest energy) *CO adsite to the bidentate-*v* configuration of *CHO, consistent with the energetic ordering reported in Table 1. For a deeper look into the bonding, the CASSCF AS natural orbitals of *CO, *CHO, and *COH at their respective preferred adsites are shown in SI Section S6, Figure S10.

We visualize in Figure 2 the relative energies (Table 1) of the different adsorbate configurations on surface-vacancy-containing Cu(111), plotting them alongside the analogous relative energetics predicted for these adsorbates on pristine Cu(111).^30^ It is immediately evident, with one exception (*CHO at hollow-3*c*), that the presence of a vacancy drives stronger site preferences than a perfect Cu(111) surface does (since orange symbols are mostly above blue symbols). Specifically, *CO binds most strongly at the atop site on both pristine and vacancy-containing Cu(111) surfaces (see blue and orange circles, lower left), with alternative CO adsites on defective Cu(111) (orange circles) less stable than similar adsites on the pristine surface (blue-filled circles). A similar vacancy-induced stabilizing trend exists for *COH and *CHO: their respective preferred hollow-*3c* and bidentate-*v/p* adsites (orange and blue squares and triangles) are each favored over other adsorption geometries more strongly on vacancy-containing rather than pristine Cu(111). Thus, vacancies appear to help pin the adsorbates to a particular site.

**Figure 2:**
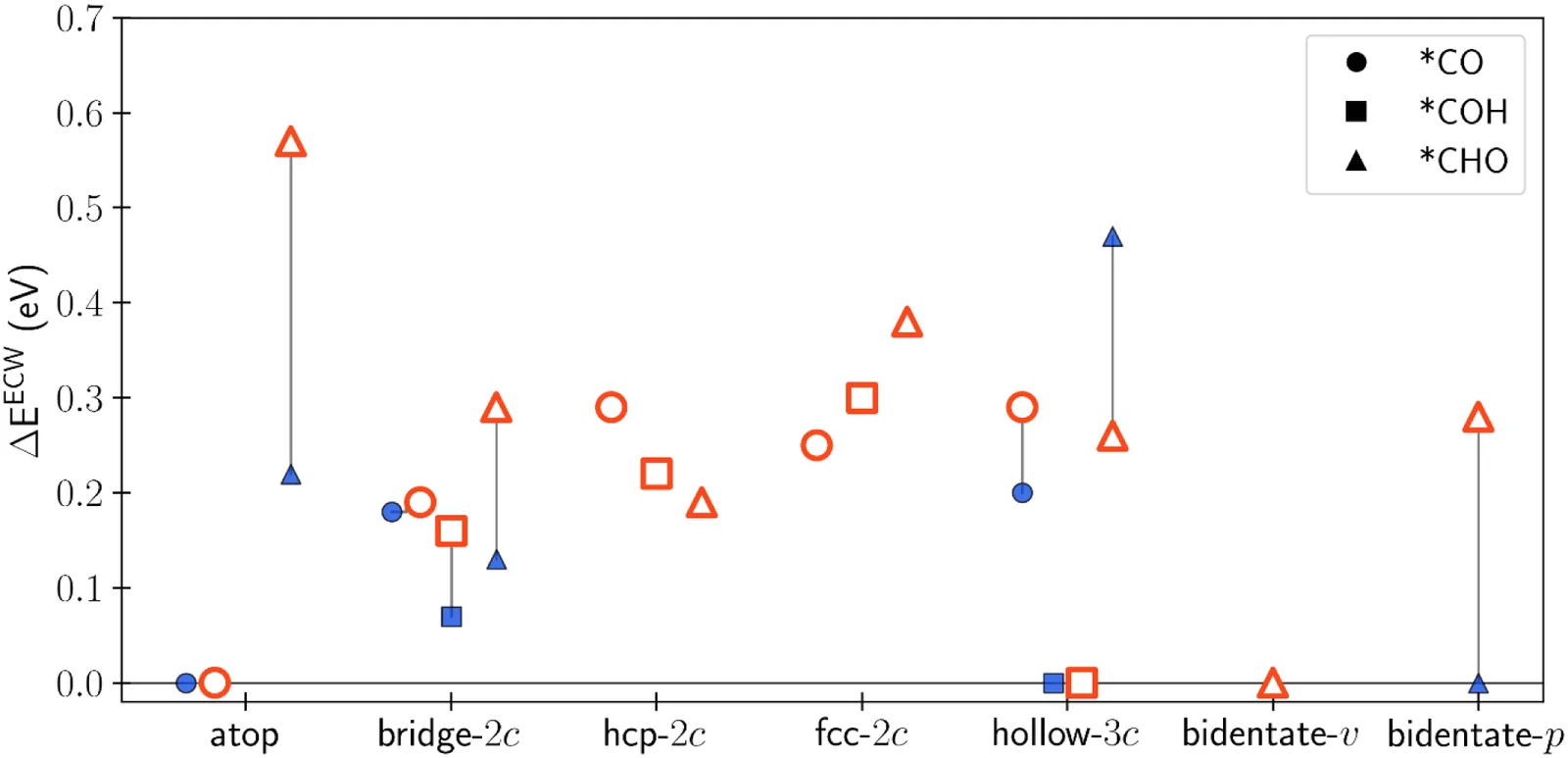
Relative ECW energies for adsites of eCO_2_R intermediates: *CO (circles), *COH (squares), *CHO (triangles), on pristine (blue solid-filled) and surface-vacancy-containing (orange unfilled) Cu(111) surfaces. ΔE^ECW^ is referenced to the lowest-energy site (set to zero) where each adsorbate/surface-type is identified by a symbol with unique color, shape, and fill. Vertical lines connect similar sites on pristine and vacancy-containing surfaces. Atomic structures for adsites on Cu(111)-vacancy are shown in SI Figure S1, with preferred adsites in Figure 1. ΔE^ECW^ are reported using ECW = emb-CASPT2 for both vacancy-containing Cu(111) (this work) and pristine-Cu(111) (taken from ref 30).

With the preferred adsorption configurations for *CO, *CHO, and *COH determined, we constructed two independent PCET pathways starting from *CO at the atop site and terminating at *CHO in bidentate-*v* and *COH in hollow-*3c*, as discussed next.

### 3.2 PCET-Mediated CO Reduction Reaction Pathways

We determined PCET MEPs for the reduction of *CO to *CHO and *COH using the CI-NEB method at the DFT-PBE-D3­(BJ) level within the [2S]-sol model, as described in Section 2.2. We compare MEPs optimized for other PW-DFT models – [1S], [1S]-sol, and [1S] with single-point implicit-solvent corrections – with [2S]-sol in SI Section S4, Figure S6. [2S]-sol produces correct long-range solvent polarization effects and appropriate surface charge densities for periodic slab systems; a physical test confirming this is provided in SI Section S2. The ISs and FSs of the MEPs are derived from the most stable adsites of the reduction reactants and products, as predicted by ECW theory (see Table 1 and Figures 1–2). The Eigen cation is located relative to the reactant *CO at a water-proton-to-CO-carbon distance R­(C–H) of 2.57 Å, with the nearest proton oriented toward the *CO (this is the IS structure after geometric relaxation with *CO fixed at *atop* as described in Section 2.2 above), along with two spectator water molecules added on the surface (see Figure 3). Structure files are provided as SI. AS natural orbitals for critical structures along the two MEPs are provided in SI Section S7, Figures S11–S15.

**Figure 3:**
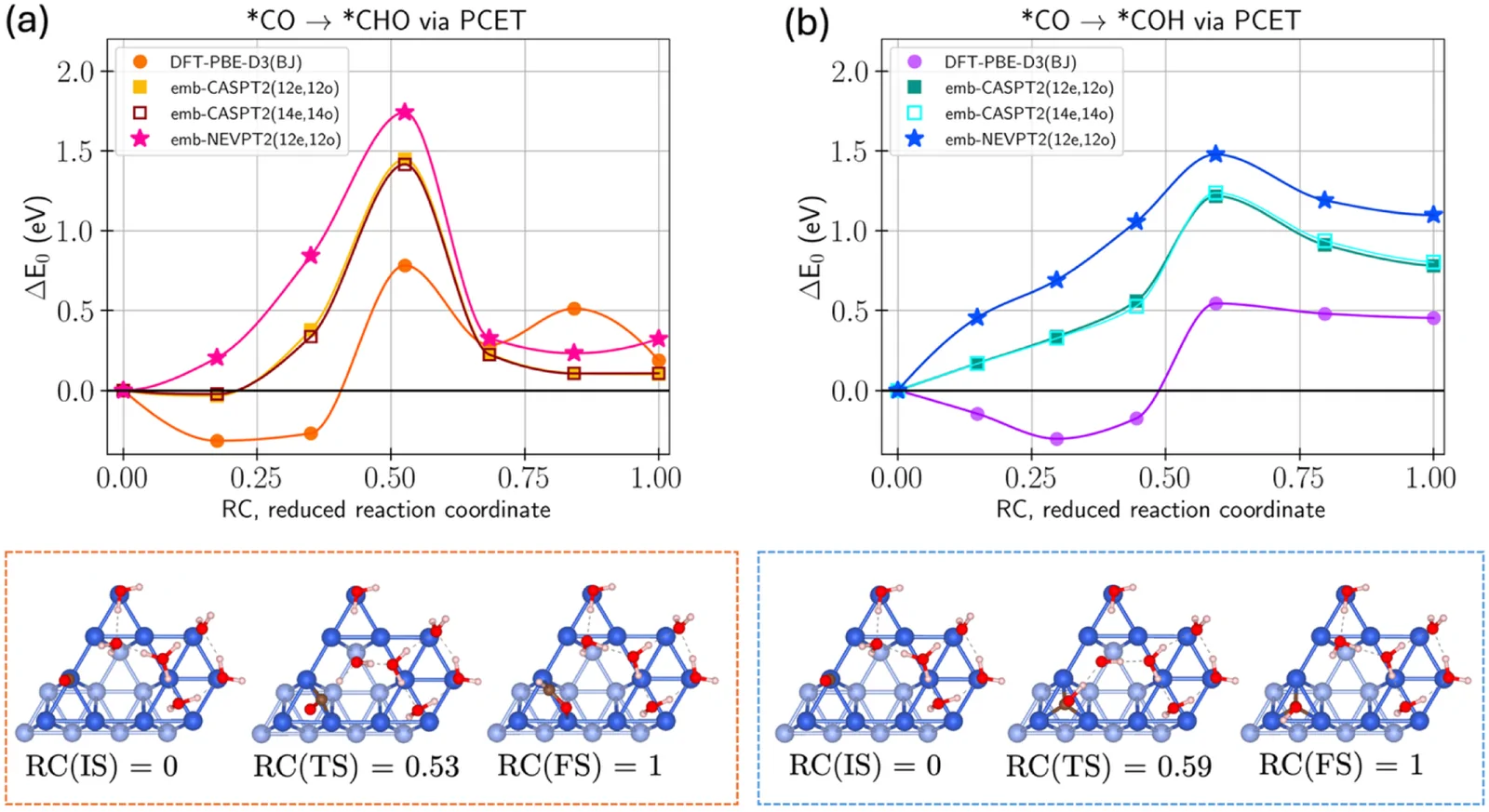
Relative internal energies (at *T* = 0 K, without ZPE corrections) for the PCET reduction pathways for (a) *CO → *CHO and (b) *CO → *COH, on Cu(111) with a nearby surface vacancy. The MEP was first optimized with CI-NEB using DFT-PBE-D3­(BJ) with continuum water ([2S]-sol model), constrained to have reactants and products in their ECW-predicted lowest-energy adsites. The ECW refinement of the MEP energetics then was performed with two active space sizes (12e,12o) and (14e,14o) in emb-CASPT2, and (12e,12o) in emb-NEVPT2. The critical structures – IS, TS, and FS – along the respective MEPs are shown in the insets below (a) and (b). See SI Section S4 for the reduced reaction coordinate (RC) definition. At the TS, the transferring proton is midway between *CO and H_3_O^+^ in (a), and already at *CO­(H) in (b). The pathway in (b) exhibits within DFT a pseudobarrier (only one soft imaginary frequency at 38 cm^–1^) with a mostly flat path onward to the FS but shows a more pronounced TS within ECW. The CASSCF natural orbitals at each critical point for (a) and (b) are displayed in SI Section S7. All energies are tabulated in SI Section S8, Table S2.

#### 3.2.1 *CO → *CHO via Reaction with an Eigen Cation in Continuum Water

Starting from CO chemisorbed at the atop site adjacent to the vacancy (reduced reaction coordinate, RC­(IS) = 0 in Figure 3a), *CO first migrates to a neighboring bridge (SI Section S4, Figure S7) similar to bridge-*2c* in Figure S1a, and then to the 3-fold hollow site similar to hollow-*3c* in Figure S1a, which is erroneously favored by DFT-PBE. This sequence of events spans the region RC < 0.53, after which *CO returns to the bridge site at the TS given by RC­(TS) = 0.53 in Figure 3a. At the TS, the Eigen cation approaches *CO, enabling long-range proton transfer to *CO via proton shuttling from the reactant H_3_O^+^ through an H_2_O adjacent to *CO, with concomitant electron transfer from the Cu surface to *CO. The FS consists of *CHO and four H_2_O molecules still in solution, along with two spectator *H_2_O. The *CHO first forms at a bridge site, then undergoes a frustrated rotation/translation toward an atop-bidentate configuration (see SI Section S4, Figure S7, images 4-5), appearing as a smaller secondary bump along the MEP, before relaxing into the more preferred bidentate-*v* configuration in the FS, shown in Figure 3a.

**Figure 4:**
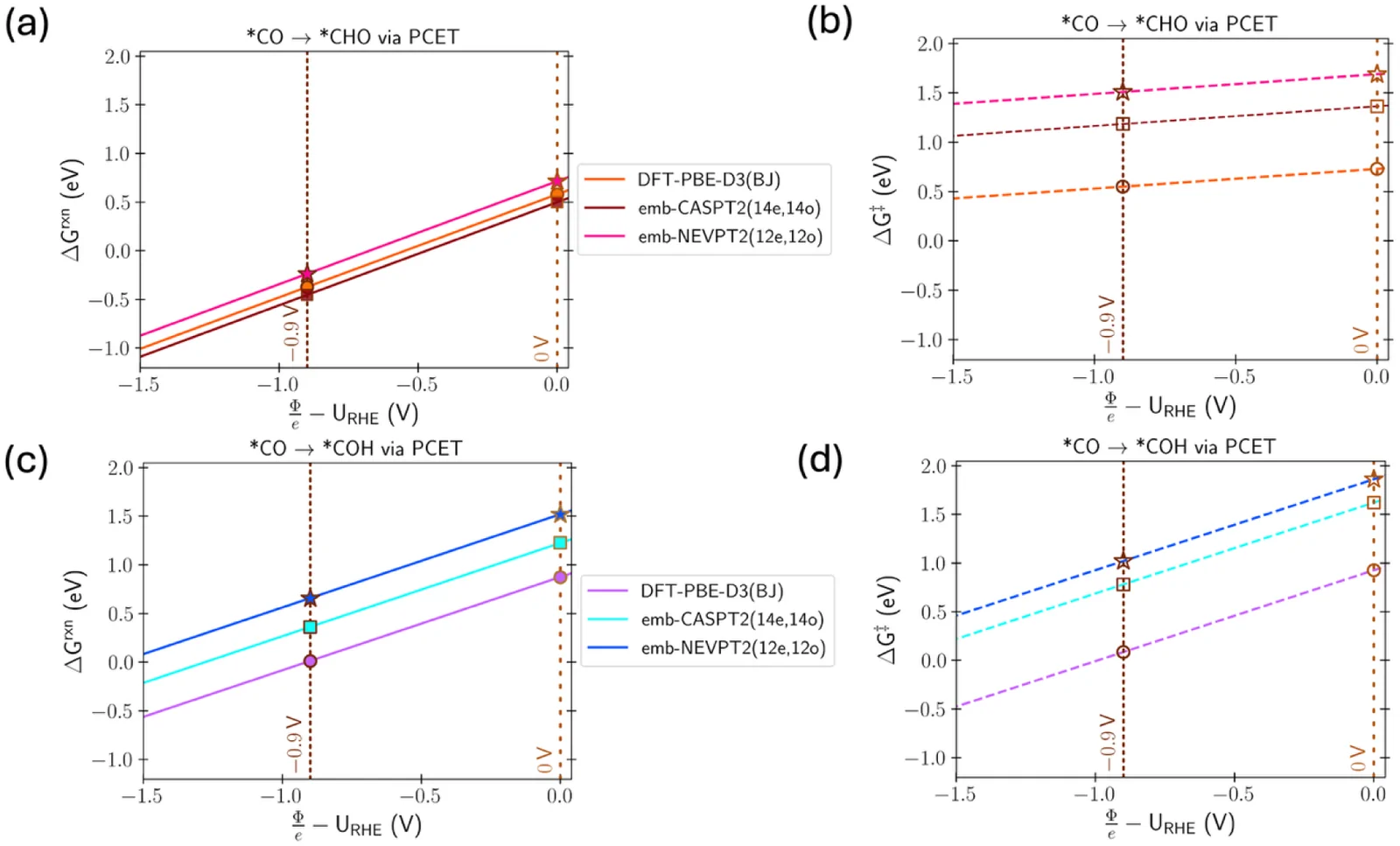
Potential-dependent reaction free energies ΔG^rxn^ (solid lines) and activation free energies ΔG^‡^ (dashed lines) for the PCET conversion of (a), (b) *CO → *CHO and (c), (d) *CO → *COH, referenced to the reversible hydrogen electrode (RHE) potential at pH = 0. Vertical dotted lines indicate electrode potentials of 0 V vs RHE (light brown, sparse dots) and −0.9 V vs RHE (dark brown, dense dots). Filled and open markers denote the intersections of ΔG^rxn^ and ΔG^‡^, respectively, with these potentials; marker shapes identify the computational method (circle: DFT-PBE-D3­(BJ); square: emb-CASPT2; star: emb-NEVPT2). Given the nearly identical (overlapping) data from emb-CASPT2­(12e,12o) and (14e,14o), we only show the latter. All free energies are tabulated in SI Section S8B, Table S6.

**Figure 5:**
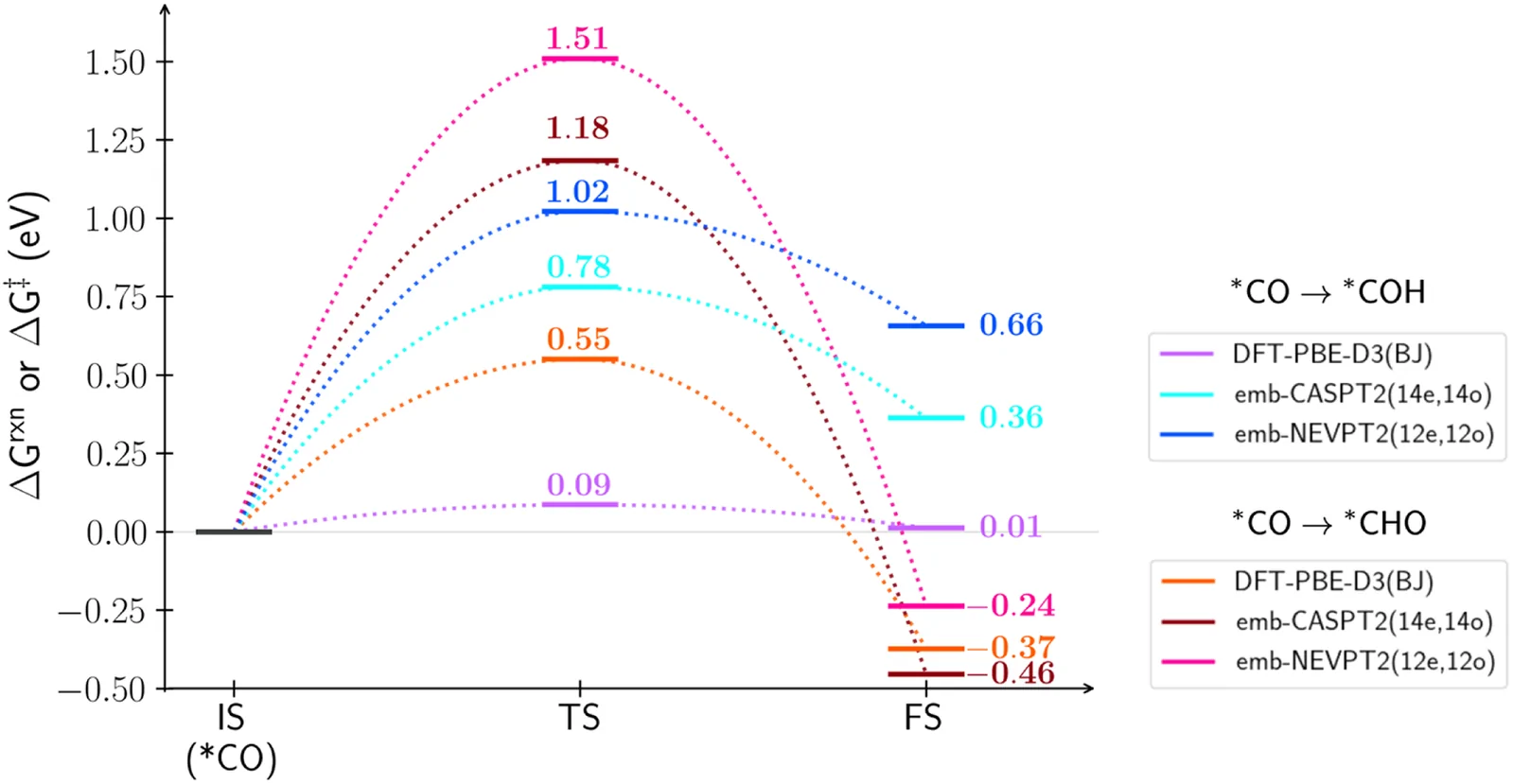
Reaction and activation free energies for *CHO and *COH formation on vacancy-containing Cu(111) at an electrode potential of $\frac{\Phi }{e}=-0.9\;V$ vs RHE at different levels of theory. The IS corresponds to *CO at an atop site adjacent to the surface vacancy. In the FS, *CHO adopts the bidentate-*v* configuration next to the surface vacancy, whereas *COH occupies the hollow-*3c* site.

The relative energies ΔE_0_ in Figure 3a are referenced to the IS corresponding to *CO at the atop site, identified as the most stable configuration in the absence of an Eigen cation (and without the corresponding excess electron in the Cu slab) by emb-CASPT2 and emb-NEVPT2 (see Table 1). This choice enables us to compare the two competing eCOR pathways on an equal footing and draw further comparisons between surface-vacancy-containing Cu(111) and pristine Cu(111).^30^ At the DFT-PBE level, the reaction energy and activation barrier for the PCET conversion of *CO to *CHO near a vacancy are 0.19 and 0.78 eV, respectively (orange curve in Figure 3a and SI Section S8, Table S2). Static ECW refinements of the energies of all DFT-optimized structures along the IS/FS-constrained MEP then were obtained. We first selected an AS of (14e,14o) from emb-CASSCF calculations, as described in Section 2.3. Applying eq 2 at the emb-CASPT2 (14e,14o) level yields a reaction energy of 0.11 eV and an activation barrier of 1.42 eV, which are respectively 0.08 eV lower and 0.64 eV higher than the corresponding DFT-PBE-D3­(BJ) values for *CHO formation (see Figure 3a). For emb-NEVPT2, the largest tractable AS was found to be (12e,12o). We therefore compared emb-CASPT2 results for (12e,12o) and (14e,14o), to assess whether any essential correlation effects are lost upon decreasing the AS size slightly. The emb-CASPT2­(12e,12o) energies very closely match those of the larger AS, yielding a reaction energy of 0.10 eV and an activation barrier of 1.45 eV, suggesting the (12e,12o) AS is sufficient. We then carried out emb-NEVPT2­(12e,12o) calculations, obtaining increased reaction and activation energies of respectively 0.32 and 1.74 eV, both significantly higher than DFT-PBE-D3­(BJ) values (Figure 3a).

The TS involves bond breaking and forming and thus is multiconfigurational by nature, as evidenced by the configuration-interaction coefficients obtained after the CASSCF cycle, where three coefficients exceed a magnitude of 0.10 with the largest being 0.93. It is well known that DFT-GGA overdelocalizes electrons due to self-interaction error, resulting in reaction barriers that are spuriously too low for electron-transfer and bond-breaking/forming processes. CASSCF and subsequent multireference PT2 methods yield the correct electron-transfer description by including exact exchange and a multiconfigurational correlation treatment of the metal–adsorbate reacting system. The latter treatment ensures that incorporation of exact exchange does not lead to known divergences of the electronic structure in metals as seen with single-configuration, uncorrelated, Hartree–Fock theory. The importance of this multiconfiguration treatment of the metal is seen in the substantial deviation from integer [2,0] occupation of the correlated Cu-4*s* orbital pair in the CASSCF wavefunction, e.g., [1.82, 0.18] at the TS; see SI Section S7. *The results in* Figure 3 *illustrate the inability of single-determinant DFT to accurately describe electron-transfer and bond-breaking/forming relative to multireference PT2 methods, with the largest deviation occurring at the TS, where electron delocalization is maximal.*

The analogous pathway on pristine Cu(111),^30^ examined using a similar but not identical modeling approach, led to an emb-CASPT2­(14e,14o) reaction energy of 0.88 eV and barrier of 1.13 eV. As noted above, the vacancy-containing Cu(111) reaction energy is 0.11 eV with a barrier of 1.42 eV at the same emb-CASPT2 level of theory for our [2S]-model. In ref 30, implicit solvation was included as a single-point correction to the MEP optimized in vacuum and the periodic DFT energy was based on a [1S]-model, so the comparison is inexact. Based on data we present in SI Section S4, Figure S6, however, the MEP following ref 30’s [1S]-model, denoted as [1S] sp-sol in Figure S6a, fortuitously agrees very well with our more rigorous [2S]-model, predicting a barrier too high by only 0.03 eV and a reaction energy too low by only 0.03 eV, enabling a robust comparison of the predictions comparing pristine and defective Cu(111) MEPs. Thus, the 0.3 eV increase in the PCET barrier suggests that creating vacancies on Cu(111) should inhibit *CHO formation. That said, the more favorable reaction energy (however, both are still endothermic) suggests that, if the barrier can be surmounted, the product *CHO will bind more favorably to the vacancy-containing surface than to pristine Cu(111).

#### 3.2.2 *CO → *COH via Reaction with an Eigen Cation in Continuum Water

The alternative *CO reduction intermediate, *COH, preferentially adsorbs at the hollow-*3c* site (*vide supra*). We therefore next examine a PCET pathway connecting *CO and *COH at their respective ECW-preferred adsites. As the Eigen cation approaches to initiate proton transfer, *CO migrates from the atop site to hollow-*3c* via the bridge-*2c* site (SI Section S4, Figure S8), corresponding to the region with RC < 0.59 in Figure 3b. At the instant of proton transfer, RC­(TS) = 0.59 in Figure 3b, CO is already located at the hollow-*3c* site rather than atop. Consequently, we see a divergence between the DFT-PBE and ECW energy profiles for RC < 0.59: DFT erroneously stabilizes *CO at the hollow site, whereas ECW corrects this behavior and identifies the atop site as its true minimum.

As in the case of *CHO formation, the CO–H bond forms via concerted proton shuttling from hydronium (H_3_O^+^) to the H_2_O molecule nearest to *CO, and onward to *CO. As shown in Figure 3b, the nearly flat potential energy surface for RC > 0.59 provides no clear TS at the DFT level. After the proton-transfer event (RC > 0.59 in Figure 3b), the relatively flat energy profile corresponds to the facile rotation of the CO–H bond away from the previously hydrogen-bonded H_2_O molecule (SI Section S4, Figure S8, images 5 and 6). At the DFT-PBE level (SI Section S8, Table S2), the reaction energy and activation barrier are 0.45 and 0.55 eV, respectively, when referenced to *CO at the atop site, indicating a kinetically viable *COH formation. Subsequent ECW refinements of the DFT-PBE MEP give reaction energies of 0.78 eV [emb-CASPT2­(12e,12o)], 0.80 eV [emb-CASPT2­(14e,14o)], and 1.10 eV [emb-NEVPT2­(12e,12o)], referenced to *CO at atop (Figure 3b). The corresponding ECW energies of the apparent TS at RC = 0.59 increase substantially to 1.22 eV [emb-CASPT2­(12e,12o)], 1.24 eV [emb-CASPT2­(14e,14o)], and 1.48 eV [emb-NEVPT2­(12e,12o)]. This pronounced difference between ECW and DFT-PBE, especially at the TS, is again attributed to the tendency of DFT-PBE to spuriously overdelocalize electrons, leading to too-facile electron transfer and bond breaking/forming.

Lastly, compared to analogous pathways on pristine Cu(111),^30^ the presence of a surface vacancy does not dramatically alter the *CO → *COH path. As in the pristine case,^30^ *COH prefers the 3-fold hollow site (hollow-3*c*) on Cu(111) even in the presence of a vacancy. *COH still coordinates with three Cu atoms on the defective surface, although one of these Cu is undercoordinated near the vacancy. Consistent with the similar binding motifs on the two surfaces, the activation barriers to form *COH from *CO by PCET on pristine Cu(111) are nearly the same as for the vacancy-containing surface, to within ∼0.1 eV: DFT-PBE-D3­(BJ) and emb-CASPT2­(14e,14o) are 0.43 and 1.15 eV, respectively, for pristine Cu(111)^30^ compared to 0.55 and 1.24 eV, respectively, for vacancy-containing Cu(111). Consequently, the presence of vacancies will not substantially alter the kinetics of the *CO → *COH path on Cu(111).

Overall, these values suggest that vacancy-containing Cu(111) may exhibit a kinetic preference for forming *COH over *CHO – a robust conclusion for all the ECW results in Figure 3 – with a barrier that is ∼0.2 eV lower to form *COH; this stands in contrast to pristine Cu(111), where the barriers to form *COH and *CHO were nearly identical, suggesting competitive formation rates for each. However, our analysis thus far has relied on constant-charge quantum simulations, wherein as electrons transfer from the electrode, the surface work function changes, implying that the electrode potential changes during the reaction. This is not physically correct, as typically electrochemistry is conducted under constant-potential conditions. Accordingly, we next convert our constant-charge energetics to free energies at constant electrode potential, to enable direct comparison with experiment.

### 3.3 Potential-Dependent Activation and Reaction Free Energies for PCET Conversion of *CO → *CHO/*COH

We first convert the above constant-charge internal energies to constant-charge Gibbs free energies of reaction (ΔG^rxn^) and activation (ΔG^‡^) (SI Section S8, Table S3). Since the MEPs were optimized in the presence of an implicit solvent, vibrational frequency analyses were carried out within the [2S]-sol PW-DFT framework defined in Section 2.2 to obtain ZPE and entropic corrections for the conversion from internal to free energies. Together, ΔG^rxn^ and ΔG^‡^, converted to constant-potential conditions, will provide a combined first-principles thermodynamic and kinetic picture of the electrocatalytic hydrogenation of CO at constant temperature, pressure, and electrode potential.

The potential dependence is introduced *a posteriori* into free energies evaluated for IS, TS, and FS structures optimized along a constant-electron DFT-MEP. Consequently, this treatment does not include any potential-dependent reorientation of the explicit water network or associated further relaxation of the proton-transfer geometry and transition state. Such geometric response can contribute to nonlinear potential dependence of electrochemical barriers.^103^ That said, our model Eigen cation (H_9_O_4_)^+^ reactant is fully relaxed in the presence of a negatively charged metal surface, so qualitatively we have a physically appropriate reactant configuration in the presence of a negative potential. As mentioned earlier, since we only consider negative potentials as in experiment, water reorientations along the path may be limited, with our simulation being a reasonable representation of the structures along the path. Full validation of these predictions awaits a future in which it is possible to conduct these extremely time-intensive simulations across a multitude of potential-dependent solvent configurations.

To this end, we employ a modified Chan−Nørskov model^32,33^ in combination with the computational hydrogen electrode (CHE) model^104^ to transform the free energies computed at a constant number of electrons to their constant-potential counterparts.^30,36,105^ In this approach, based on a capacitor-like approximation of the electric double layer, we establish a linear form for the potential dependence of reaction and activation free energies,

3 $${\Delta G}_{a\to b}\left(\Phi \right)=\Delta n_{e}\left[a\to b\right]\frac{\Phi }{e}+\Delta G_{a\to b}\left(\Phi =0\right)$$

4 $${\Delta G}_{a\to b_{TS}}^{‡}\left(\Phi \right)=\Delta n_{e}\left[a\to b_{TS}\right]\frac{\Phi }{e}+{\Delta G}_{a\to b_{TS}}^{‡}\left(\Phi =0\right)$$

for reactant *a*, product *b*, and transition state *b*_*TS*_. The slope of the linear fit is given by the number of electrons transferred (Δ*n_e_*), obtained using a Bader charge partitioning scheme in PW-DFT (described in SI Section S8A), with the vertical axis intercept given by

5 $$\Delta G_{a\to b}\left(\Phi =0\right)=\left[G_{b}\left(\Phi_{b}\right)-G_{a}\left(\Phi_{a}\right)\right]\;-\Delta n_{e}\left[a\to b\right]\left(\frac{\Phi_{a}+\Phi_{b}}{2e}\right)$$

6 $${\Delta G}_{a\to b_{TS}}^{‡}\left(\Phi =0\right)=\left[G_{b_{TS}}\left(\Phi_{b_{TS}}\right)-G_{a}\left(\Phi_{a}\right)\right]\;-\Delta n_{e}\left[a\to b_{TS}\right]\left(\frac{\Phi_{a}+\Phi_{b_{TS}}}{2e}\right)$$

for ΔG^rxn^ and ΔG^‡^, respectively.

Because of the presence of continuum water in the [2S]-sol model, the work functions of Cu(111) surfaces computed directly within PW-DFT are referenced to the bulk of the implicit solvent rather than to vacuum. We denote this quantity as *δϕ*, such that *δϕ* = *Φ* – *ϕ_sol_*, where Φ is the “absolute” work function of the electrode in vacuum (to be determined) and *ϕ_sol_* is the work function of continuum water, which we derived using the CHE (see SI Section S8). Using eqs 3–6 in conjunction with the CHE framework,^32,33,36^ wherein $H^{+}+e^{-}↔\frac{1}{2}H_{2}$ , and accordingly G­(H^+^ + e^–^) is replaced by $\frac{1}{2}G\left(H_{2}\right)$ , we first obtain the linear dependence of the reaction and activation free energies on *δϕ* (see SI Section S8B, Tables S3–S6 for the computed values). Once we have *δϕ*, we simply add *ϕ*_*sol*_ to it to obtain Φ. Finally, we reference Φ to the measured reversible hydrogen electrode (RHE) potential at pH = 0 $\left(U_{\mathrm{RHE}}{|}_{\mathrm{pH}=0}=4.281\;V\right)$ ,^106^ i.e., we subtract 4.281 V from our computed Φ. ΔG^rxn^ and ΔG^‡^ as a function of electrode potential vs RHE potential (denoted $\frac{\Phi }{e}-U_{\mathrm{RHE}})$ for the PCET conversion of *CO to *CHO and *COH are shown in Figure 4.

The experimental onset potential for methane and ethylene formation on Cu(111) is −0.9 V vs RHE.^107,108^ In Figure 4, vertical lines correspond to $\frac{\Phi }{e}-U_{\mathrm{RHE}}=0\;V$ and $\frac{\Phi }{e}-U_{\mathrm{RHE}}=-0.9\;V$ (equivalently, $\frac{\Phi }{e}=0$ and −0.9 V vs RHE, respectively); their intersections with the free energy vs $\frac{\Phi }{e}-U_{\mathrm{RHE}}$ plots yield the free energies of formation and activation of *CHO and *COH at those respective potentials vs RHE. Distinct marker shapes used for the intersection points denote the different levels of theory employed. The nearly identical emb-CASPT2 results obtained with AS of (12e,12o) and (14e,14o) lend credence to use of the smaller AS for emb-NEVPT2, which is computationally prohibitive at (14e,14o) within the current Molpro implementation. The sensitivity of NEVPT2 to the choice and size of AS must be left for future work.

For the PCET reduction of *CO → *CHO shown in Figure 4a and b, DFT-PBE-D3­(BJ) reaction and activation free energies at 0 V vs RHE are +0.58 eV (orange filled circle) and +0.73 eV (open circle), respectively. Incorporation of ECW corrections via emb-CASPT2 slightly lowers the reaction free energy to +0.50 eV (brown filled square), while raising the activation barrier to +1.36 eV (open square); emb-NEVPT2 predicts a reaction free energy of +0.72 eV (magenta filled star) and an activation barrier of +1.69 eV (open star). The PCET conversion of *CO → *COH at 0 V vs RHE yields a reaction free energy of +0.87 eV (purple filled circle) along with an activation barrier of +0.93 eV (open circle) at the DFT-PBE level, as shown in Figure 4c and d, respectively. Subsequent ECW corrections with emb-CASPT2 further increase the reaction free energy to +1.22 eV (cyan filled square) with a barrier of +1.62 eV (open square), while emb-NEVPT2 furnishes a reaction free energy of +1.52 eV (blue filled star) with a barrier of +1.86 eV (open star). At $\frac{\Phi }{e}=0\;V$ vs RHE, both *CO reduction pathways require surmounting substantial kinetic barriers; however, *CHO formation consistently exhibits a lower activation barrier and is thermodynamically more favorable than the competing *COH pathway across all levels of theory. A more meaningful assessment of the kinetics, however, is obtained at the experimentally relevant onset potential of $\frac{\Phi }{e}=-0.9\;V$ vs RHE. The corresponding values obtained by linear extrapolation of ΔG^rxn^ and ΔG^‡^ to this onset potential are also displayed in Figure 5.

At an applied potential of $\frac{\Phi }{e}=-0.9\;V$ vs RHE, DFT-PBE-D3­(BJ) predicts *CHO formation to be exergonic by −0.37 eV with a kinetically accessible barrier of +0.55 eV, whereas *COH formation is nearly thermoneutral at 0.01 eV and kinetically facile, with a barrier of only 0.09 eV (Figure 5). Upon inclusion of ECW corrections, emb-CASPT2 and emb-NEVPT2 predict *CHO formation to remain exergonic by −0.46 eV and −0.24 eV, respectively, albeit with significantly higher activation barriers of +1.18 eV and +1.51 eV. In contrast, ECW predicts *COH formation to be endergonic with reaction free energies of 0.36 eV (emb-CASPT2) and 0.66 eV (emb-NEVPT2), and corresponding activation barriers of 0.78 eV (emb-CASPT2) and 1.02 eV (emb-NEVPT2). The corresponding reverse barriers are 0.42 eV (emb-CASPT2) and 0.36 eV (emb-NEVPT2) for *COH, compared with 1.64 eV (emb-CASPT2) and 1.75 eV (emb-NEVPT2) for *CHO. The substantially lower kinetic barrier for *COH formation indicates a switch in selectivity toward the COH pathway for the first *CO PCET step at $\frac{\Phi }{e}=-0.9\;V$ vs RHE. In fact, this inverted preference occurs at much less negative electrode potentials starting at ∼−0.3 (−0.2) V vs RHE within emb-CASPT2 (emb-NEVPT2). Even though emb-CASPT2 and emb-NEVPT2 methods differ quantitatively by up to ∼0.2–0.3 eV, they are qualitatively consistent, i.e., they predict the same thermodynamic and kinetic ordering. At −0.9 V vs RHE, the *COH barrier is lower than the *CHO barrier by 0.40 eV with emb-CASPT2 and by 0.49 eV with emb-NEVPT2. Both methods also predict *CHO formation to be exergonic and *COH formation to be endergonic at this potential. Thus, the method sensitivity does not alter the predicted kinetic preference for *COH formation.

In comparison, for eCOR on pristine Cu(111),^30^ DFT-PBE-D3­(BJ) predicted a TS-free but exergonic *COH formation at $\frac{\Phi }{e}=-0.9\;V$ vs RHE, contrary to experiments reporting the onset of methane production at this potential.^107,108^ This discrepancy was resolved therein^30^ by ECW calculations on pristine Cu(111), which revealed comparable activation barriers of +0.58 eV (for *COH) and +0.56 eV (for *CHO), with *CHO formation being slightly thermodynamically favored, though still endergonic (+0.20 eV), as shown in Figure 6. While pristine Cu(111) shows an equal propensity for eCOR to *COH and *CHO, the presence of a vacancy gives a ∼0.40 eV lower barrier to form *COH compared to *CHO, at emb-CASPT2­(14e,14o). Note that ref 30’s pristine Cu charge computations were done differently, see SI Section S8, which makes direct comparison to the present results imperfect. However, taken altogether, these comparisons do indicate that the introduction of vacancies alters the potential energy landscape of eCO_2_R on Cu(111), in turn modifying the preferred reduction pathways. The ∼0.40 eV lower activation barrier associated with the PCET conversion of *CO →*COH at −0.9 V vs RHE, as revealed by ECW treatments, gives it a kinetic advantage over the competing *CHO pathway and points to an overall vacancy-driven selectivity of Cu(111) toward *COH formation. The subsequent, and likely faster, steps that consume *COH (either coupling^34–36^ or further hydrogenation) will limit back reaction to *CO despite the low reverse barrier.

**Figure 6:**
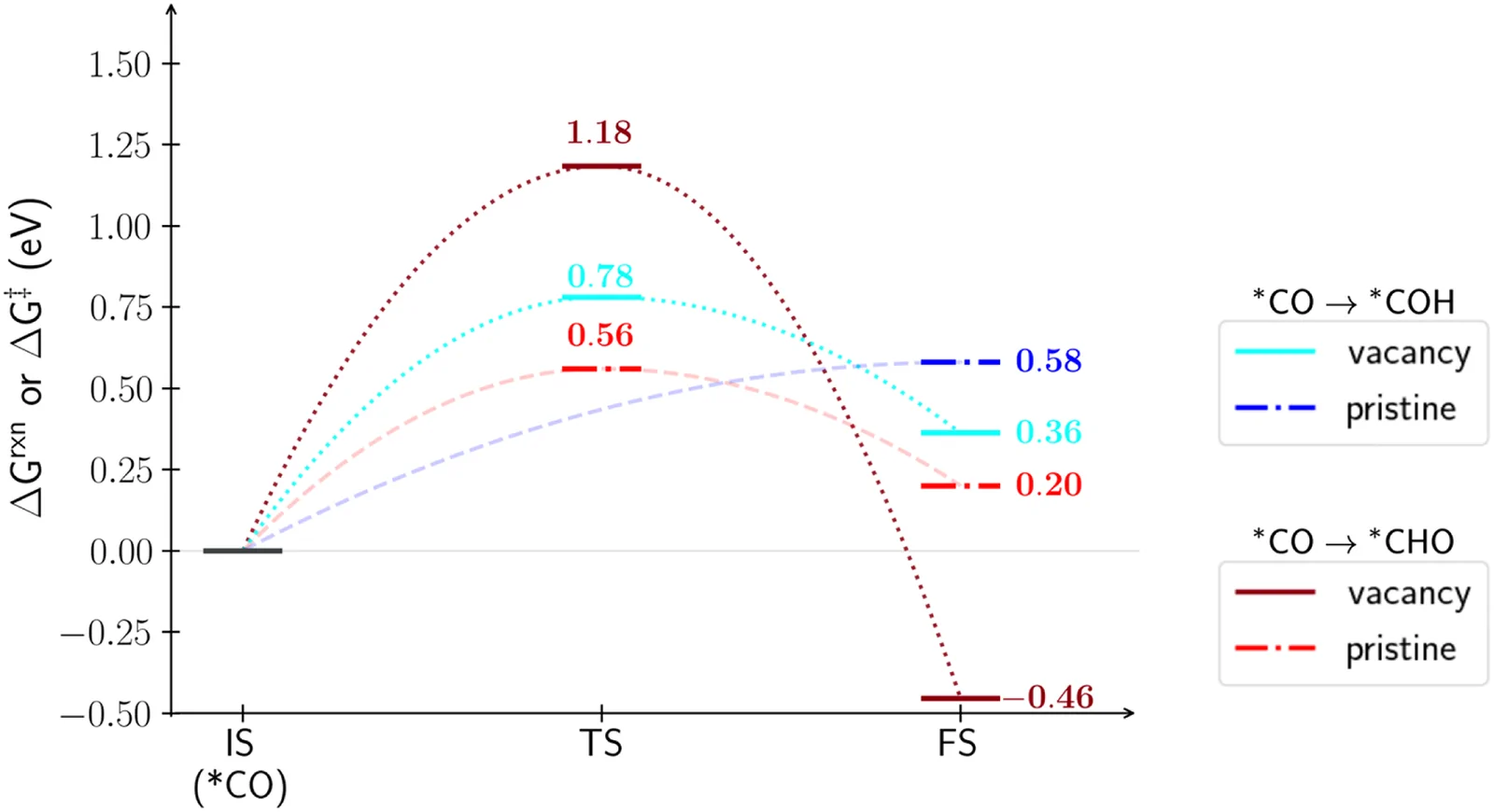
eCOR on vacancy-containing vs pristine Cu(111) surfaces: reaction and activation free energies for *CHO and *COH formation at an electrode potential of $\frac{\Phi }{e}=-0.9\;V$ vs RHE at emb-CASPT2­(14e,14o). Results for pristine Cu(111) were taken from ref 30.

## 4 Summary and Conclusions

An ECW-guided comparison of pristine and vacancy-containing Cu(111) surfaces reveals that the presence of surface vacancies affects the MEPs traversed by the primary eCO_2_R intermediates, as confirmed by our CI-NEB analyses of the competing PCET routes, *CO → *CHO and *CO → *COH. The electrochemical hydrocarbon production rate (methane and ethylene) on Cu(111) has been observed to be potential dependent,^107,108^ implying direct charge transfer from the Cu electrode to the surface-bound intermediates is involved in the rate-determining step. Our group’s earlier ECW investigations on pristine Cu(111) demonstrated that a PCET mechanism – rather than a surface-bound H-atom transfer – is consistent with this potential dependence.^30^ In the present work, we extended this higher-level electronic-structure treatment to a refined PCET model determining the effect of surface vacancies on eCOR. Following a preliminary DFT-PBE screening, we systematically improved the electronic description by incorporating exact exchange and correlation effects via emb-CASSCF and subsequent multireference second-order perturbation theory.

After transforming the free energies from constant charge to constant applied potentials via a capacitor model, the experimentally relevant applied potential Φ/e = −0.9 V vs RHE at which Cu(111) begins to form hydrocarbons is most worthy of analysis (Figures 5 and 6). At this potential, DFT-PBE-D3­(BJ) predicts exergonic *CHO formation (−0.37 eV) with an activation free energy barrier of 0.55 eV, while *COH formation is found to be thermoneutral (0.01 eV) with a negligible barrier of 0.09 eV. ECW theory confirms that *CHO formation is exergonic, with reaction free energies of −0.46 eV (emb-CASPT2) and −0.24 eV (emb-NEVPT2), but with significantly higher activation barriers of 1.18 eV (emb-CASPT2) and 1.51 eV (emb-NEVPT2) compared to DFT-PBE-D3­(BJ). By contrast, ECW theory finds *COH formation to be endergonic by 0.36 eV (emb-CASPT2) and 0.66 eV (emb-NEVPT2) with kinetic barriers of 0.78 eV (emb-CASPT2) and 1.02 eV (emb-NEVPT2), considerably lower than the competing *CHO pathway. Ultimately, higher-level CW theory indicates that Cu(111) containing surface vacancies kinetically favors *COH formation over *CHO at eCO_2_ R-relevant potentials, thereby promoting selectivity toward the *COH pathway at the first *CO PCET step. Thus, our ECW-based combined thermodynamics and kinetics investigation of vacancy-containing Cu(111) surfaces indicates *COH will preferentially form, unlike on pristine Cu(111), where both *CHO and *COH are predicted to form and thus complicate subsequent redox and C–C-coupling chemistry.^34^ Thus, a roughened Cu(111) surface containing vacancies is likely to follow kinetics more similar to that found for Cu(100), where *COH is also the dominant reduction precursor formed.

## Supplementary Material





## Notes

Atomic coordinates of *CO, *COH, and *CHO adsorption structures on vacancy-containing Cu(111); periodic slab structures along the *CO to *CHO and *CO to *COH PCET minimum-energy paths; and corresponding embedded-cluster structures used for ECW calculations, in VASP/POSCAR and XYZ formats (ZIP)
DFT-PBE-D3­(BJ) adsorption-site structures and relative energies of *CO, *COH, and *CHO; evaluation and selection of the implicit-solvent slab model; electrostatic-potential profiles and CI-NEB minimum-energy paths for the *CO-to-*CHO and *CO-to-*COH PCET reactions; DFET embedding-potential optimization; emb-CASSCF active-space natural orbitals and occupations for adsorbates and critical structures along the MEP; Bader charge-transfer analysis, work-function calibration, and constant-charge-to-constant-potential transformation of reaction and activation free energies (PDF)
