# Supplementary material for: How Surface Vacancies Alter Electrocatalytic CO2 Reduction Pathways on Cu(111): Insights from Embedded Correlated Wavefunction Theory
Source: J Phys Chem C Nanomater Interfaces. 2026 Oct 14;130(43):14857–72. doi: 10.1021/acs.jpcc.6c03598 (PMC13726464; doi:10.1021/acs.jpcc.6c03598)
Supplement: Supplementary file 1 [file jp6c03598_si_002.pdf]

Supporting Information for

How Surface Vacancies Alter Electrocatalytic CO_2 Reduction Pathways on Cu(111): Insights from Embedded Correlated Wavefunction Theory

Vidushi Sharma,[1] John Mark P. Martirez,[1] and Emily A. Carter[1,2,3*]

[1]Applied Materials and Sustainability Sciences, Princeton Plasma Physics Laboratory, Princeton, NJ 08540-6655, USA

[2]Department of Mechanical and Aerospace Engineering, Princeton University, Princeton, NJ 08544-5263, United States

[3]Andlinger Center for Energy and the Environment and Program in Applied and Computational Mathematics, Princeton University, Princeton, NJ 08544-5263, USA

*eac@princeton.edu

Table of Contents

S1. SCANNING ADSORPTION SITES FOR ECO_2R INTERMEDIATES WITH PW-DFT FOR ECW ASSESSMENT 2

S2. SIMULATION DETAILS 3

A. SETTING CAVITY SURFACE TENSION PARAMETER $\tau = 0$ IN VASPSOL FOR PERIODIC SLABS 3

B. CRITERION FOR CHOOSING [2S]-SOL MODEL IN PW-DFT WITH IMPLICIT SOLVATION 5

S3. ELECTROSTATIC POTENTIALS OF INITIAL, TRANSITION, AND FINAL STATES OF ECO_2R PATHWAYS VIA PCET 7

S4. ECO_2R MINIMUM ENERGY PATHS FROM DFT-PBE-D3(BJ): CI-NEB 8

S5. DENSITY FUNCTIONAL EMBEDDING THEORY: OPTIMIZING THE EMBEDDING POTENTIAL, Vemb 10

S6. ACTIVE SPACES FOR *CO, *COH, AND *CHO 11

S7. ACTIVE SPACES OF CRITICAL STRUCTURES ALONG THE MEPS 12

A. *CO → *CHO VIA PCET 13

B. *CO → *COH VIA PCET 14

S8. TRANSFORMATION FROM CONSTANT CHARGE TO CONSTANT POTENTIAL FREE ENERGIES 15

A. DETERMINATION OF Δne FROM BADER CHARGE PARTITIONING[8, 9] WITHIN THE [2S]-SOL MODEL 16

B. ABSOLUTE WORK FUNCTION Φ, WITHIN THE [2S]-SOL MODEL 17

SUPPLEMENTARY REFERENCES 20

S1. Scanning adsorption sites for eCO_2R intermediates with PW-DFT for ECW assessment

Fig. S1 shows the candidate adsorption sites investigated for CO_2 reduction intermediates *CO, *COH, and *CHO on Cu(111) near a surface vacancy. All configurations correspond to local minima, as confirmed via vibrational frequency analyses of the adsorbates and the topmost Cu layer, which reveal no modes with imaginary frequencies above 100i cm^{-1}.

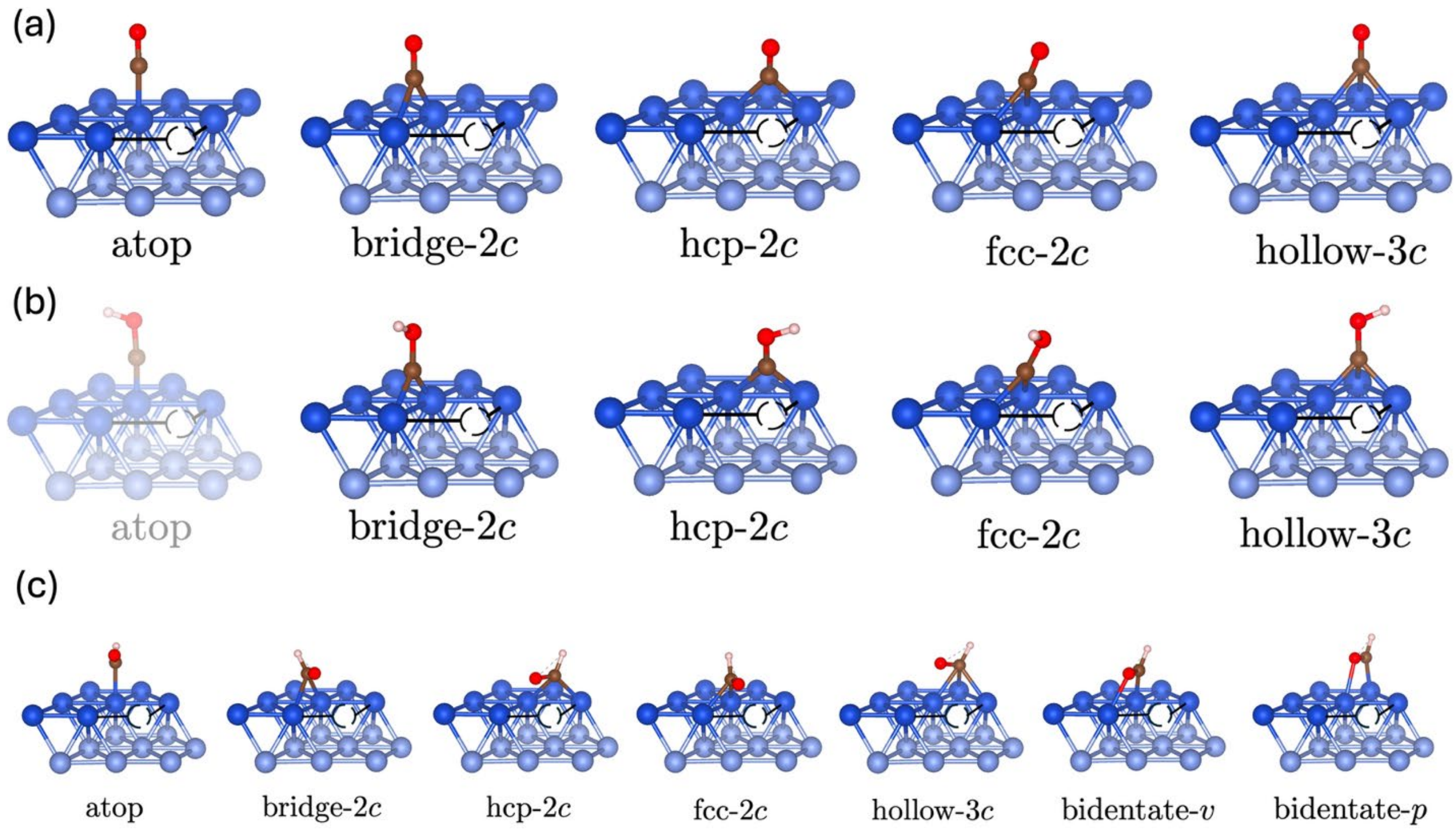

Fig. S1: DFT-PBE-D3(BJ)–optimized adsorption geometries for (a) *CO, (b) *COH, and (c) *CHO on Cu(111) near a surface vacancy (side views). Here we show only the molecular geometries of adsorbates after translating them onto the embedded clusters (Cu_{16} for *CO and Cu_{15} for *COH and *CHO) used in ECW calculations (see Computational Methodology, Sec. II*c*). The surface vacancy corresponds to the missing top-layer Cu atom at the front edge of the cluster (marked by a dashed circle). '*2c*' and '*3c*' refer to two-fold and three-fold coordination, respectively. '*v*' refers to the bidentate adsite next to the vacancy while '*p*' indicates a bidentate adsite farther away from the vacancy, mimicking a pristine-surface-like site. Relative energies for the structures at the PW-DFT and ECW levels appear in Table 1 in Sec. III(A) of the main text. Atop *COH was tested but found not to be a minimum (non-negligible imaginary frequencies) at the PW-DFT level and therefore was excluded from further analysis. Atoms are indicated by spheres colored as follows, Cu: blue, C: brown, O: red, H: white, with the subsurface Cu atoms faded for clarity.

Table S1. DFT-PBE-D3(BJ) adsite energies for eCO_2R intermediates, *CO, *COH, and *CHO: internal energy (E_0), zero-point energy (ZPE), relative adsite energy (Δ) without (ΔE_0) and with ZPE (ΔE_0+ZPE), referenced to the most preferred site for each adsorbate. Inclusion of ZPE changes the relative adsite energies by a maximum of 0.008 eV for *CO, 0.019 eV for *COH, and 0.030 eV for *CHO.

*CO	E_0 (eV)	ZPE (eV)	ΔE_0 (eV)	$\Delta(E_0$+ZPE) (eV)
**atop**	-331.367	0.575	0.154	0.157
**bridge-*2c***	-331.514	0.573	0.006	0.007
**hcp-*2c***	-331.505	0.566	0.016	0.010
**fcc-*2c***	-331.521	0.572	0	0
**hollow-*3c***	-331.453	0.563	0.067	0.059
***COH**				
**bridge-*2c***	-333.790	0.871	0.133	0.147
**hcp-*2c***	-333.697	0.869	0.226	0.237
**fcc-*2c***	-333.723	0.877	0.200	0.219
**hollow-*3c***	-333.923	0.857	0	0
***CHO**				
**atop**	-334.222	0.827	0.275	0.253
**bridge-*2c***	-334.318	0.825	0.179	0.156
**hcp-*2c***	-334.465	0.842	0.032	0.025
**fcc-*2c***	-334.467	0.828	0.030	0.010
**hollow-*3c***	-334.263	0.818	0.233	0.203
**bidentate-*v***	-334.497	0.849	0	0
**bidentate-*p***	-334.249	0.840	0.248	0.239

S2. Simulation details

A. Setting cavity surface tension parameter $\tau = 0$ in VASPsol for periodic slabs

For the initial state of *CO surrounded by the Eigen cation on Cu(111) with a surface vacancy, here we compare the *xy*-plane-averaged electrostatic (Hartree + ionic) potentials ($\langle V_H + V_{ion}\rangle$) from the [1S] vacuum and [1S]-sol implicit water models in Figs. S2(a) and (b). Since τ is an effective surface-tension parameter entering the cavitation contribution of the continuum solvent model,[1]

its default value is empirically parameterized to reproduce molecular solvation energetics rather than extended metal/solution interfaces.[2] Setting the default (0.525 meV/Å^2) surface tension parameter for the solvation cavity in VASPsol τ to zero in [1S]-sol retains the relevant effect, namely the electrostatic/electrolyte screening response of the continuum solvent, eliminates numerical noise caused by the inappropriate cavitation contribution (see Fig. S2(a)) and gives a smooth bulk potential, as shown in Fig. S2(b). That said, this [1S]-sol model has one other problem. In Fig. S2(c), we plot the linear *xy*-plane-integrated charge density difference $\Delta\rho(z)$ between [1S] and [1S]-sol models computed for the charge-neutral system (i.e., the system in which the Eigen cation's charge is balanced by addition of an electron to the metal surface). The adsorbate side of the slab exhibits physically reasonable fluctuations in $\Delta\rho$ due to the interaction of the adsorbed species with the water continuum. However, the other side of the slab (with no adsorbates) shows spurious charge localization in the form of a tiny blip. Integrating $\Delta\rho$ between the cyan-dashed lines in Fig. S2(c), we have

$$\int_{z_1}^{z_2} \Delta\rho(z)\, dz = +0.31\, e^- ,$$

indicating that this unphysical charge localization is not merely an artifact of a numerical parameter in VASPsol but, in fact, merits further investigation and improvements to the model.

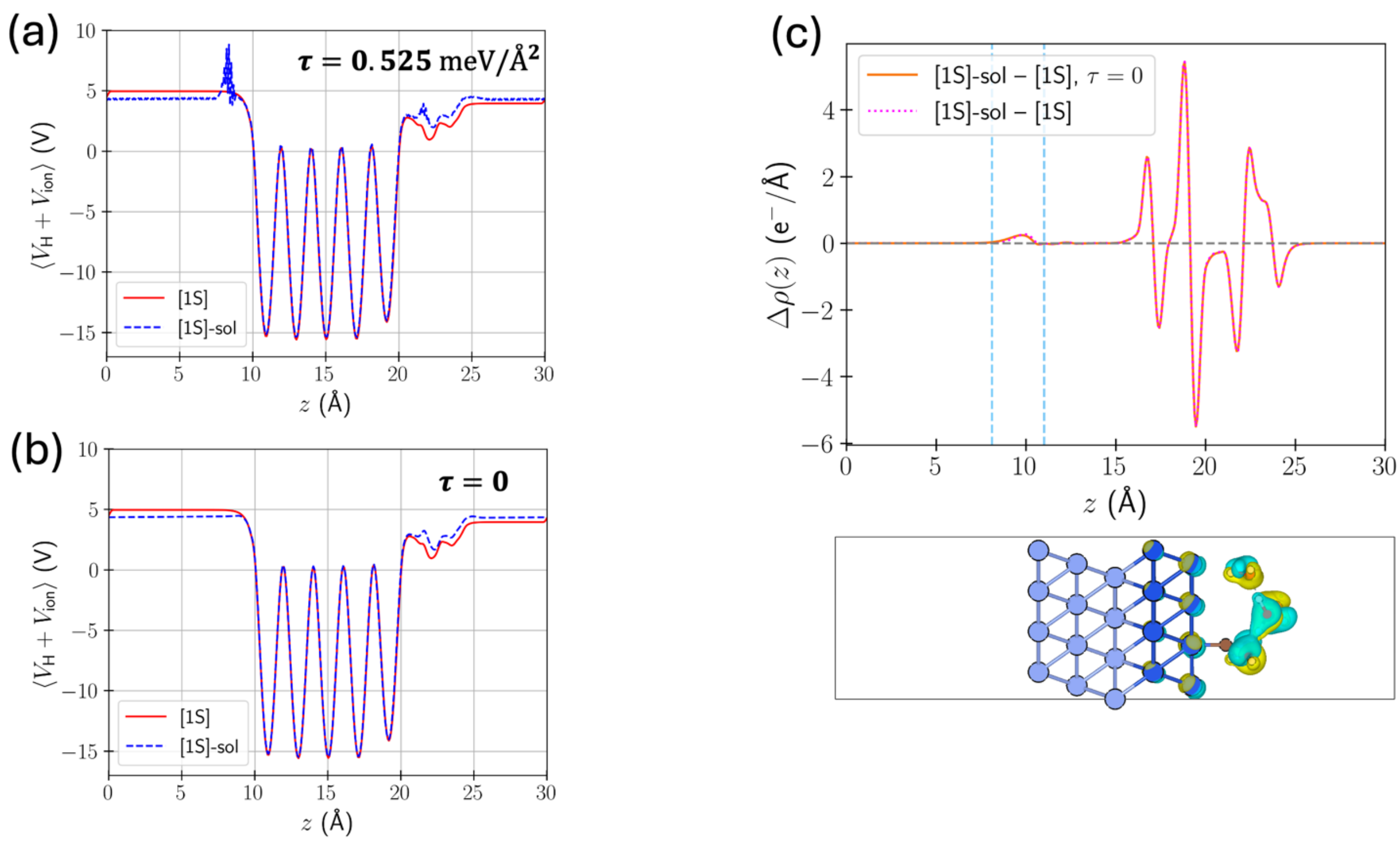

Fig. S2: *CO+$H_9O_4^+$ on one side of the Cu(111) slab with a surface vacancy: xy-plane-averaged local electrostatic (Hartree + ionic) potential $\langle V_H + V_{ion} \rangle$ with (a) default $\tau = 0.525$ meV/Å^2, and (b) $\tau = 0$, used in the implicit solvent model [1S]-sol. [1S] denotes a slab in vacuum with dipole-field corrections applied. (c) xy-plane-integrated charge-

density difference ($\Delta\rho$) between [1S]-sol and [1S] models for the charge-neutral system (see text above). The corresponding $\Delta\rho$ isosurface level = ±0.03 Å^{-3} is shown in the atomic structure below the charge-density difference plot.

B. Criterion for choosing [2S]-sol model in PW-DFT with implicit solvation

The effect of spurious charge localization in the [1S]-sol model is further enhanced upon charging the system. In Fig. S3(a) and (b), we respectively show $\langle V_H + V_{ion}\rangle - E_F$ and $\rho(z)$ for a charged system (q electrons added beyond the neutralizing first electron discussed in Sec. S2(A)) consisting of *CO+$H_9O_4^+$ on Cu(111) with a surface vacancy. In Fig. S3(c), the xy-plane-integrated density differences $\Delta\rho$ between charged and uncharged systems for various q's show an enhanced localization of the added charge on the other side of the Cu electrode. To circumvent this increasingly unphysical localization of charge, we use a [2S]-model, which by construction yields symmetric charge localization due to the adsorbates placed on both sides of the slab, as shown in Fig. S4.

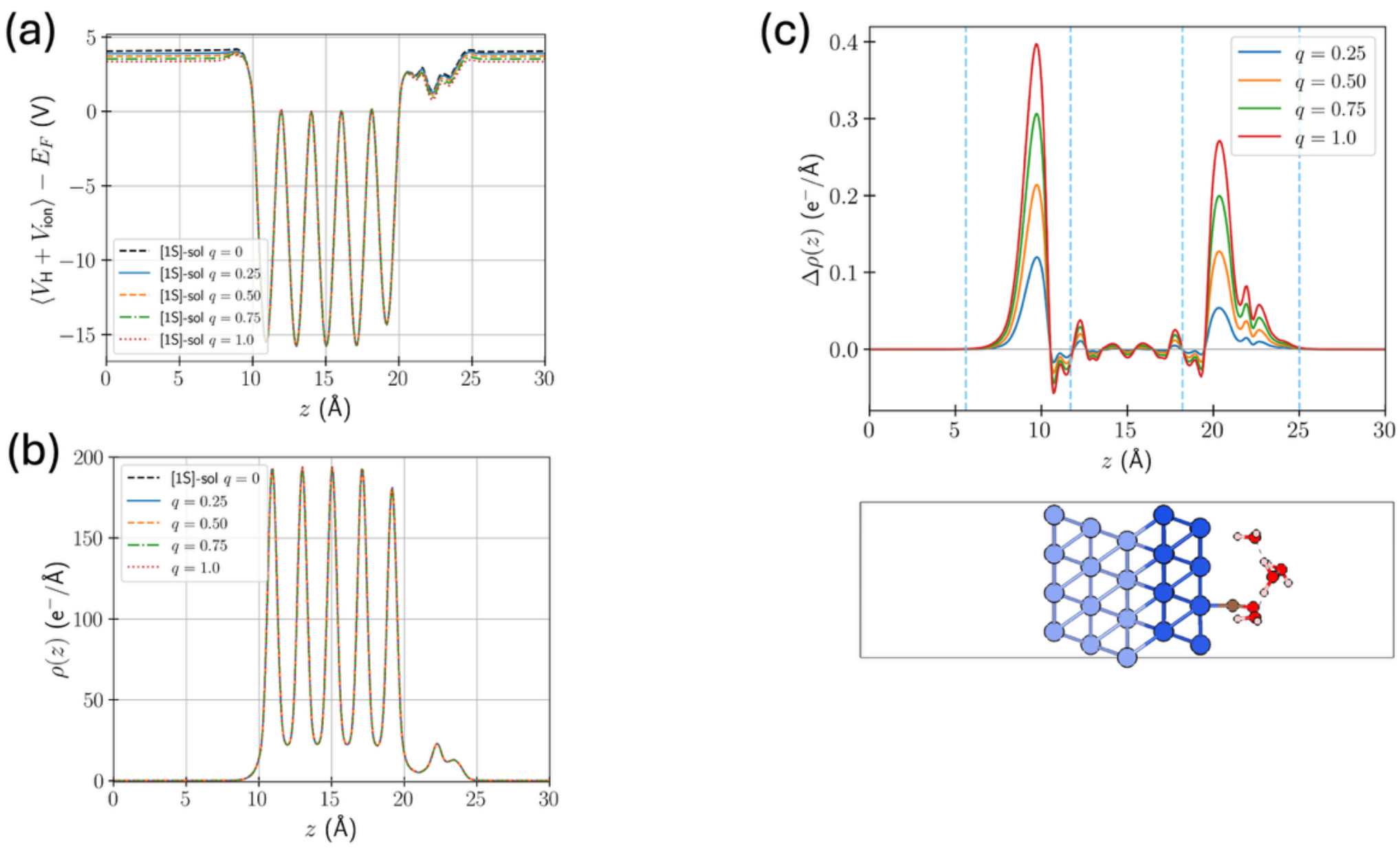

	$\int_{z_1}^{z_2} \Delta\rho(z)\,dz$	
q	q_L	q_R
0.25	0.17	0.08
0.50	0.30	0.20
0.75	0.42	0.33
1.0	0.55	0.45

Fig. S3: [1S]-sol model with $\tau = 0$: *CO+$H_9O_4^+$ on Cu(111) with a surface vacancy, and incremental charge (q electrons) added to the system. (a) xy-plane-averaged local electrostatic (Hartree + ionic) potential $\langle V_H + V_{ion}\rangle$ shifted by the Fermi level (E_F), and (b) xy-plane-integrated charge density $\rho(z)$ for q-charged systems. $\rho(z)$ is normalized such that $\int \rho(z)\,dz$, recovers the total number of electrons in the system, that is, 'NELECT' in VASP. (c) xy-plane-integrated charge density difference ($\Delta\rho$) between charged and uncharged (neutral) systems in [1S]-sol.

The [2S]-sol model yields an equal charge distribution at both adsorbate/slab interfaces (Fig. S4); hence, for all PW-DFT calculations, we employ the [2S]-model and use a normalizing factor of $\frac{1}{2}$ to report the energetics.

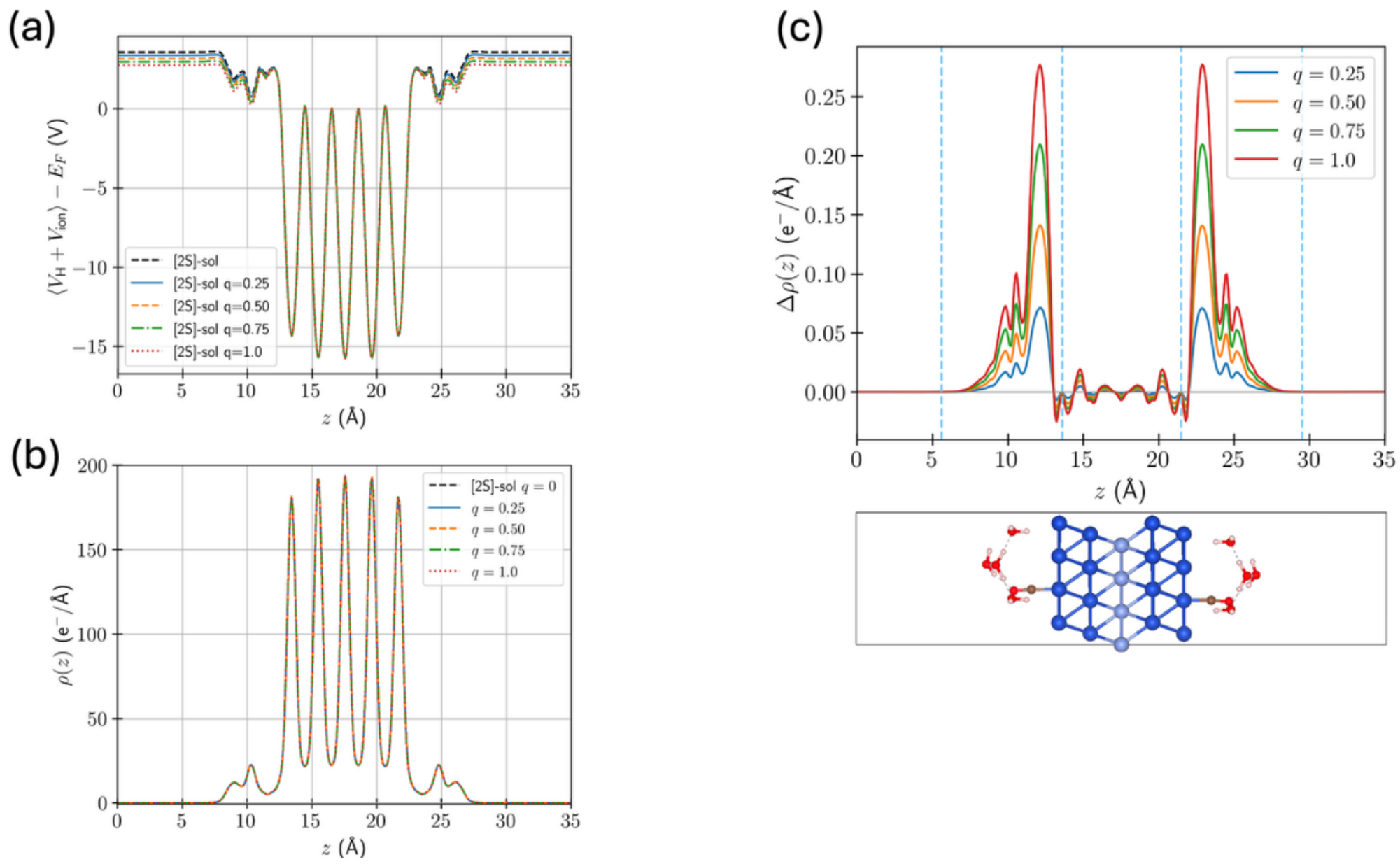

q	$\int_{z_1}^{z_2} \Delta\rho(z)\,dz$ q_L	q_R
0.25	0.13	0.13
0.50	0.25	0.25
0.75	0.38	0.38
1.0	0.50	0.50

Fig. S4: [2S]-sol model with $\tau = 0$: *CO+$H_9O_4^+$ on Cu(111) with a surface vacancy, and incremental charge (q electrons) added to the system. (a) xy-plane-averaged local electrostatic (Hartree + ionic) potential $\langle V_H + V_{ion} \rangle$ shifted by the Fermi level (E_F), (b) xy-plane-integrated charge density $\rho(z)$ for q-charged systems. (c) xy-plane-integrated charge density difference ($\Delta\rho$) between charged and uncharged (neutral) systems in [2S]-sol.

S3. Electrostatic potentials of initial, transition, and final states of eCO_2R pathways via PCET

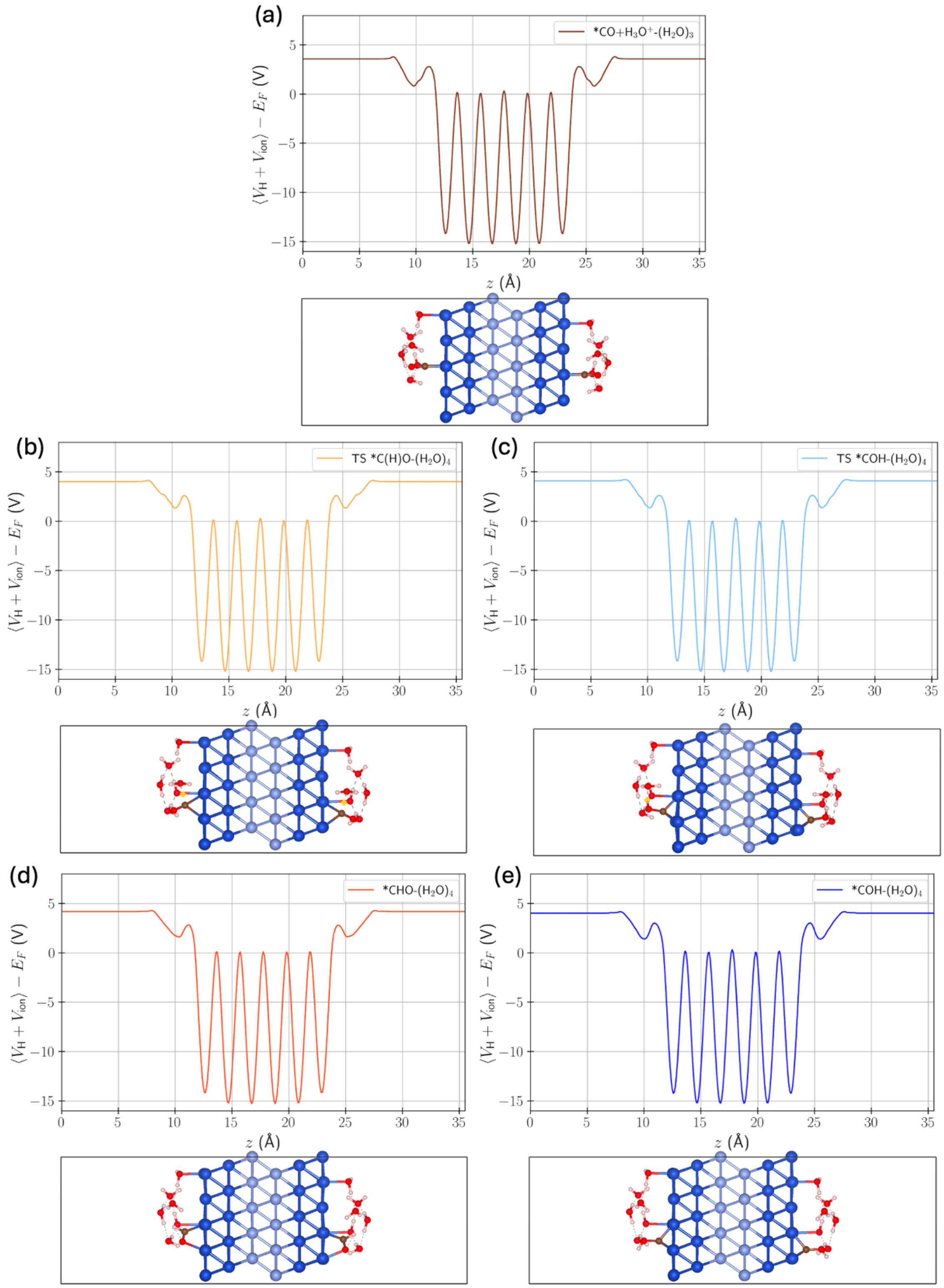

Fig. S5: xy-plane–averaged electrostatic potential for the (a) initial state (IS): *CO+$(H_3O)^+$-$(H_2O)_3$; (b) transition state (TS): *C(H)O-$(H_2O)_4$; (c) TS: *COH-$(H_2O)_4$; (d) final state (FS): *CHO-$(H_2O)_4$; and (e) FS: *COH-$(H_2O)_4$. Panels (a), (b), and (d) show the local electrostatic (Hartree + ionic) potential $\langle V_H + V_{ion} \rangle$ referenced to the Fermi level (E_F), along the *CO → *CHO PCET pathway, while panels (a), (c), and (e) show the analogously referenced $\langle V_H + V_{ion} \rangle$ along the *CO → *COH pathway. Atomic structures for each state in the [2S]-sol model are shown below the $\langle V_H + V_{ion} \rangle$ profiles; with the transferred proton at (c) TS highlighted in yellow. The Fermi level is set to zero. The two central layers of the Cu(111) slab are fixed in their equilibrium bulk positions to set a bulk-like boundary condition for the surface layers, while the two outer layers on each side and the adsorbates and species in solution are allowed to relax, except for the two adsorbed spectator waters and the reactant CO's lateral coordinates, as described below in Sec. S4 and the caption of Fig. S6; this model is denoted as *2| 2 | 2*. All [2S]-sol models consist of 148 Cu atoms and 2 spectator H_2O molecules, in addition to the species listed in (a) and (b), on each side of the Cu(111) slab.

S4. eCO_2R minimum energy paths from DFT-PBE-D3(BJ): CI-NEB

When charting the minimum energy path (MEP) of the PCET reactions, we fixed the two adsorbed spectator H_2O molecules in their optimized IS configuration along the entire MEP, including the FS. This is because if said spectator waters were allowed to relax in the FS, they desorb and combine with the FS water cluster. Therefore, without fixing said molecules, the reaction and activation free energy change would include spurious solvent reorganization that would otherwise not be present had we included a full explicit liquid water model. We explored alternative placements of the continuum-solvated water cluster, but none prevented this additional rearrangement. Beyond these two fixed water molecules, all other water-derived species - the Eigen cation in the IS, the resulting four-water cluster in the FS, and the corresponding species all along the reaction path - were relaxed along the MEP.

The reaction coordinate (*rc*) is given by the cumulative distance between structural images optimized along the MEP while accounting for the periodic boundary conditions,

$$rc(n) = \sum_{j=0}^{n-1} \sqrt{\sum\nolimits_{i=1}^{N_a} \left(R_{i,j+1} - R_{i,j}\right)^2}\,; \quad n \in \{1, \dots, N_I\},$$

where N_I is the number of images, and the IS and FS are indexed as $n = 0$ and $n = 6$, respectively. $R_{i,j}$ is the Cartesian position of atom i in image j for a total number of atoms, N_a. The reduced reaction coordinate (RC) is rc normalized by its maximum value and hence dimensionless.

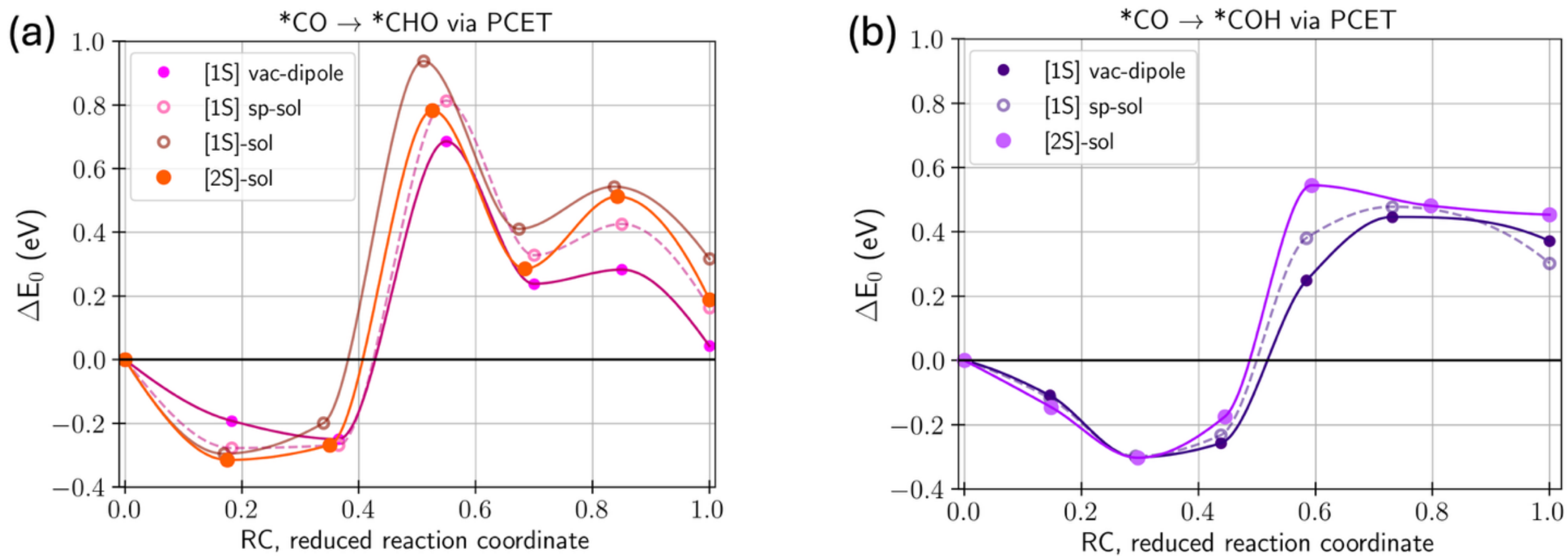

Fig. S6: MEPs on the vacancy-containing Cu(111) surface for PCET conversion of (a) *CO → *CHO, and (b) *CO → *COH, computed using PW-DFT with the climbing-image nudged elastic band (CI-NEB) method. Prior to MEP optimization, the IS and FS were structurally relaxed, including relaxation of all Cu metal atoms in the outer two layers of the metal slab (inner layers of Cu are fixed to represent bulk metal), all adsorbates, and the Eigen cation reactant in the IS and the corresponding waters in the FS, except that the adsorbed spectator waters were held fixed at their IS-optimized coordinates and the lateral (x, y) coordinate of C in *CO in the IS was constrained to be at its ECW-predicted lowest-energy adsite position (atop), which is different from its DFT-preferred structure, as discussed in Sec. III(A) of the main text. The notation [1S] and [2S] correspond to adsorbates on one side and two sides of the Cu(111) slab with a surface vacancy, respectively. 'vac-dipole' refers to PW-DFT calculations in vacuum with dipole-field corrections; 'sp-sol' indicates single-point implicit-solvent corrections applied to CI-NEB pathways obtained in vacuum; and 'sol' refers to MEPs optimized in the presence of implicit solvation.

After benchmarking the [1S]-based models against [2S]-sol for the pathway in Fig. S6(a), we performed CI-NEB calculations for [2S]-sol and the more computationally tractable [1S] models for the pathway in Fig. S6(b), omitting [1S]-sol to conserve computational resources. The [2S] and [1S] models differ by at most 0.09 (0.15) eV at the TS (FS) in Fig. S6(b).

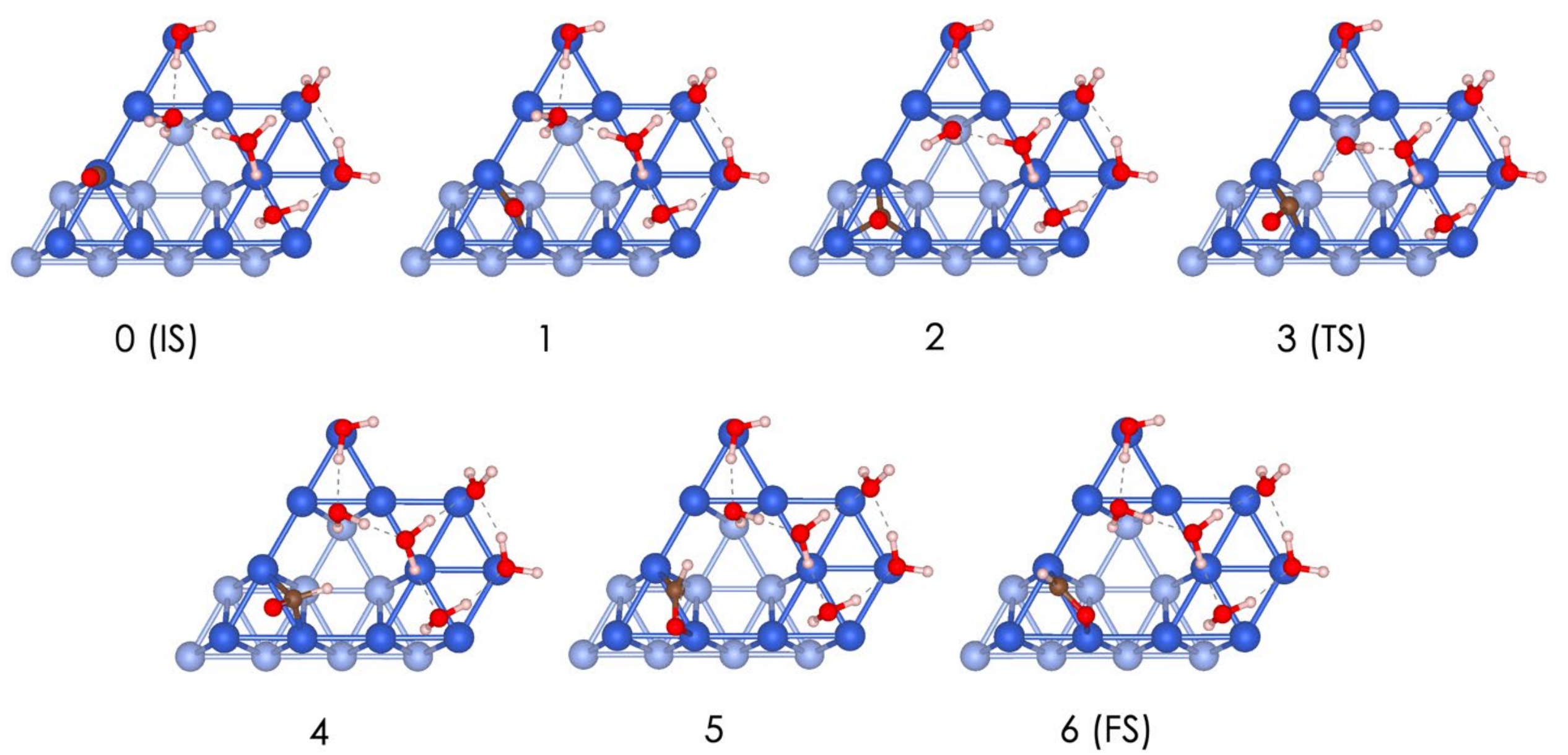

Fig. S7: DFT-PBE-D3(BJ)–optimized geometries (“images,” top view) along the MEP for the PCET conversion *CO → *CHO, with IS and FS comprising ECW-predicted lowest-energy adsite structures, *CO: atop, and *CHO: bidentate-*v*. The MEP was generated using a [2S]-sol model, with symmetric adsorbates on both sides of the vacancy-containing Cu(111) slab, following which the ECW refinement of the energies of the IS/FS-constrained MEP structures was conducted for one set of adsorbates on the emb-Cu_{19} cluster as shown. Atoms are indicated by spheres colored as follows, Cu: blue, C: brown, O: red, H: white with the subsurface Cu atoms faded for clarity.

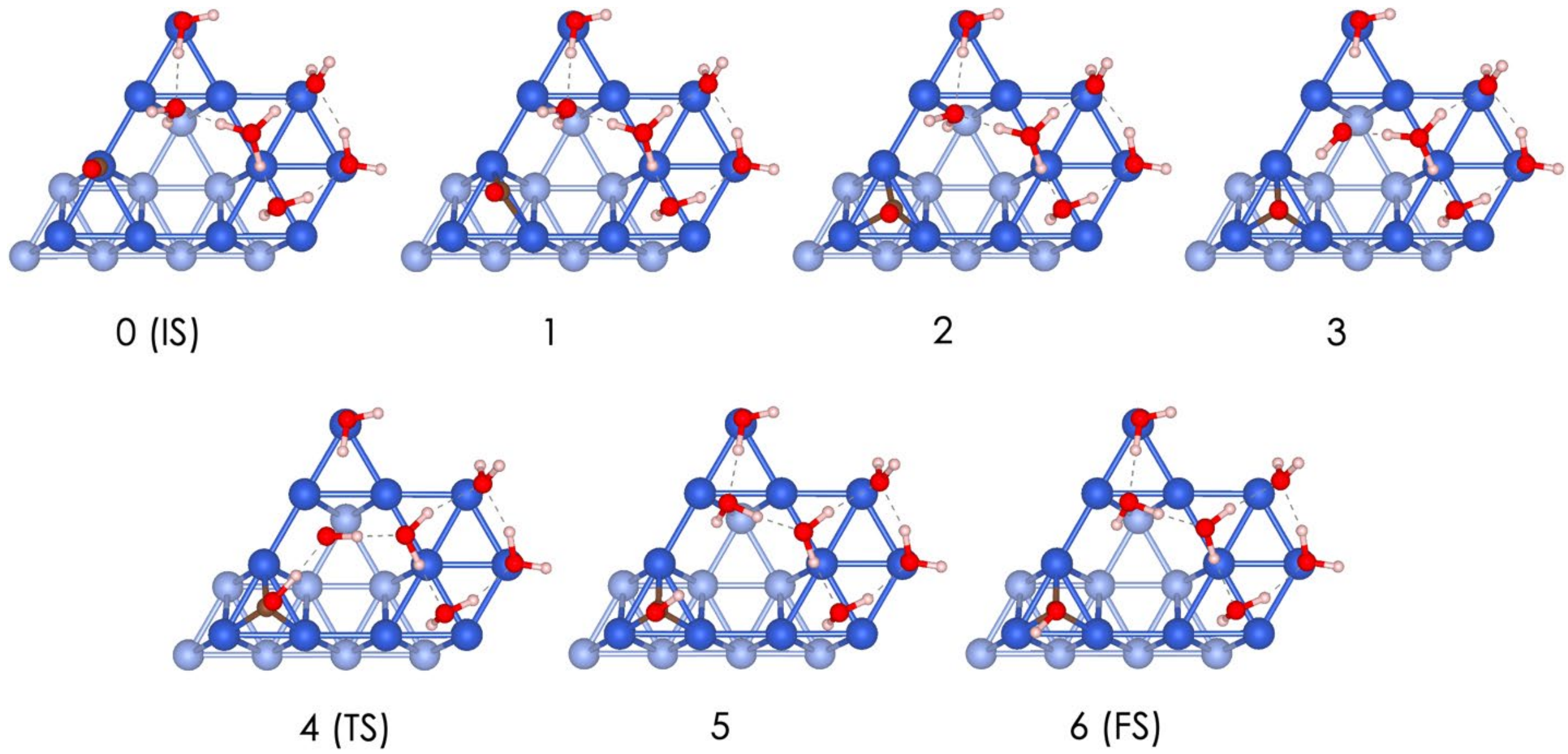

Fig. S8: DFT-PBE-D3(BJ)–optimized geometries (“images,” top view) along the MEP for the PCET conversion *CO → *COH, with IS and FS comprising ECW-predicted lowest-energy adsite structures, *CO: atop, and *COH: hollow-*3c*. The MEP was generated using a [2S]-sol model, with symmetric adsorbates on both sides of the vacancy-containing Cu(111) slab, following which the ECW refinement of the energies of the IS/FS-constrained MEP structures was conducted for one set of adsorbates on the emb-Cu_{19} cluster as shown. Atoms are indicated by spheres colored as follows, Cu: blue, C: brown, O: red, H: white with the subsurface Cu atoms faded for clarity.

S5. Density Functional Embedding Theory: Optimizing the embedding potential, V_{emb}

Using density functional embedding theory,[3-6] we partition the system into a *cluster* and its extended *environment* and then determine an embedding potential (V_{emb}) that captures their mutual interaction at the DFT-PBE level. Here, V_{emb} is obtained using an optimized effective potential (OEP) technique applied to the two subsystems. Specifically, we define an extended Wu-Yang functional $\left(\mathrm{W}[V_{emb}](\boldsymbol{r})\right)$ in terms of the total energy functionals of the subsystems in the presence of V_{emb}, as given in Eq. (1) of the main text; maximizing $\mathrm{W}[V_{emb}](\boldsymbol{r})$ recovers charge conservation in the system. The number of electrons in each subsystem is fixed upon partitioning, leading to a unique V_{emb} shared by both subsystems that is exact within the DFT XC approximation chosen. In practice, the optimization is carried out by minimizing $-\mathrm{W}_p$ with a penalty function p using an L-BFGS algorithm, as illustrated by examples in Fig. S9. The penalty term regularizes the OEP procedure, ensuring a smooth and numerically stable V_{emb}.[5]

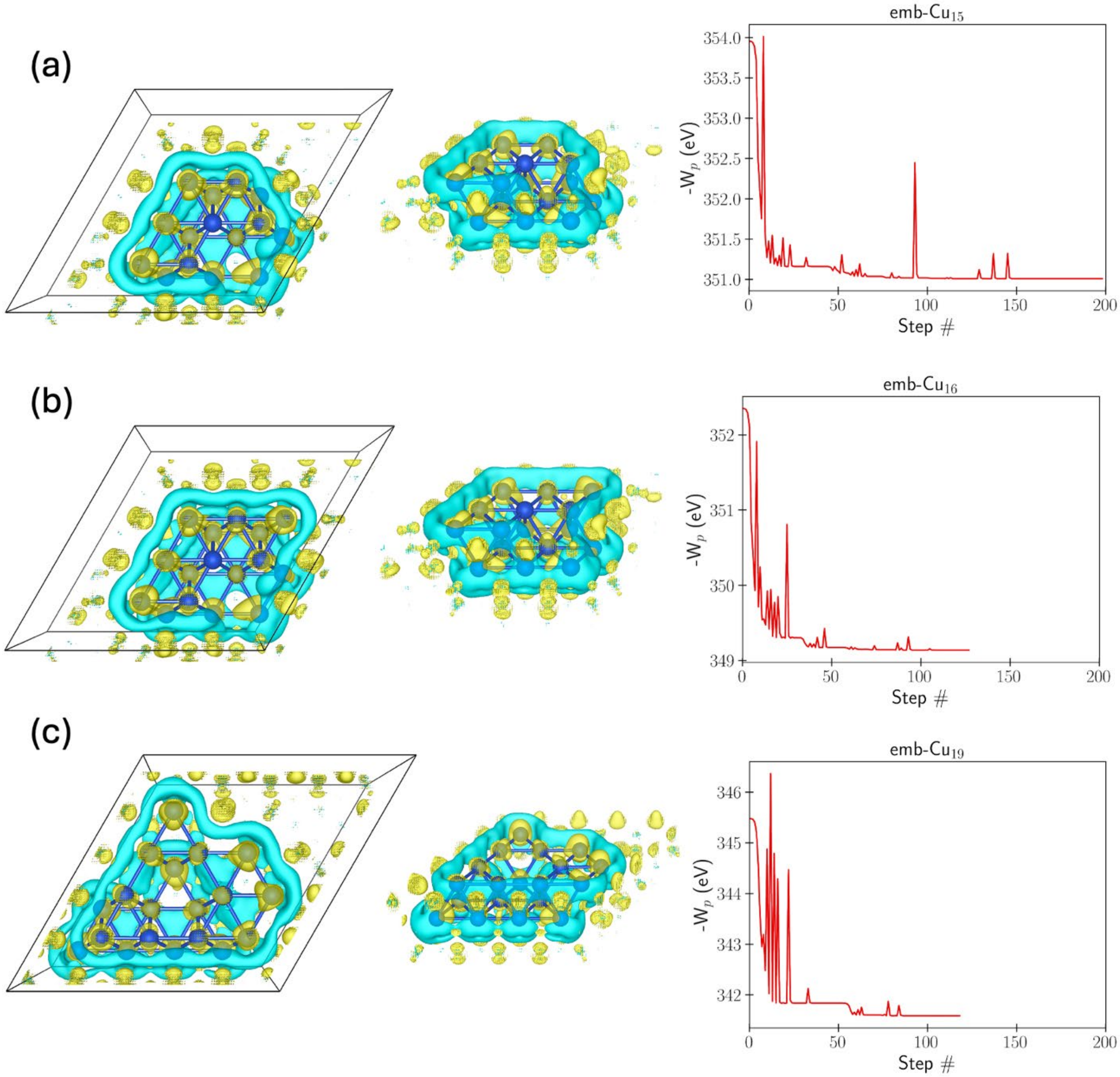

Fig. S9: Embedding potential (V_{emb}) generated for an ECW-assessment of possible adsorption sites of: (a) COH/CHO on an emb-Cu_{15} cluster with an environment of Cu_{84}. (b) CO on emb-Cu_{16} environ-Cu_{83}, and the PCET conversion of: (c) *CO → *COH and *CO → *CHO on an emb-Cu_{19} cluster with an environment of Cu_{80}. V_{emb}'s are indicated by yellow- and cyan-shaded isosurfaces with values of ±1 V. $-W_p$ (extended Wu-Yang functional, Eq. (1) in the main text, with smoothing penalty function)[5] is plotted versus optimization step (step #), illustrating its minimization with respect to $V_{emb}(\boldsymbol{r})$ and the convergence to a unique V_{emb} for the given cluster-environment.

S6. Active spaces for *CO, *COH, and *CHO

We perform a spatially localized refinement of the exchange-correlation (XC) description of adsorbates on embedded clusters subject to an embedding potential derived from DFET, with XC refined first within CASSCF, followed by second-order perturbation theory (CASPT2 and NEVPT2). The first step is to select a chemically intuitive CASSCF active space. The emb-

CASSCF wavefunctions (specifically, the canonical orbitals obtained by diagonalizing the Fock operator) provide the optimal zeroth order wavefunction inputs to multireference second-order perturbation theory calculations, emb-CASPT2 and emb-NEVPT2. The natural orbitals of the emb-CASSCF wavefunctions (obtained by diagonalizing the density matrix to obtain orbital occupations) for *CO, *COH, and *CHO in their preferred adsites are shown in Fig. S10, along with their natural orbital occupations.

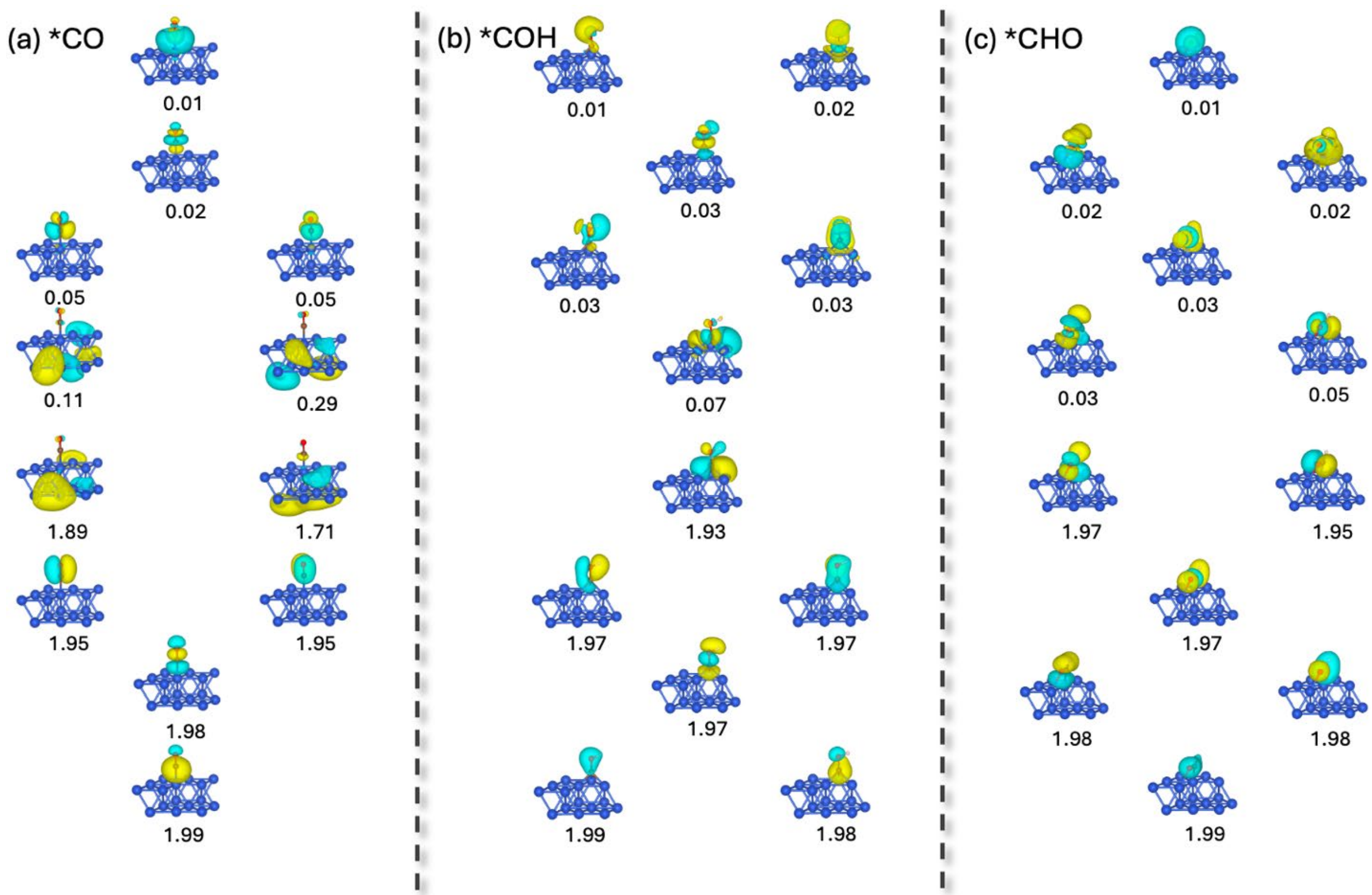

Fig. S10: emb-CASSCF (12e,12o) AS natural orbitals for eCO_2R intermediates at their preferred adsites near a surface vacancy on Cu(111): (a) *CO (atop), (b) *COH (hollow-*3c*), (c) *CHO (vacancy-adjacent bidentate-*v*). The AS used initial guesses obtained by merging (6e,6o) from *CO and (6e,6o) from emb-Cu_{16} cluster in (a), and (7e,7o) from *COH/*CHO and (5e,5o) from emb-Cu_{15} cluster in (b), (c), followed by orbital rotations in and out of the AS to select the desired initial guess CAS orbital character. The corresponding geometries without the orbitals are shown in Fig. 1 of the main text. Atoms are shown as spheres colored as follows, Cu: blue, C: brown, O: red, H: white; cyan- and yellow-colored isosurfaces (at level = ±0.03 a.u.) represent the natural orbitals, with their corresponding occupation numbers given below each orbital.

S7. Active spaces of critical structures along the MEPs

Fig. S11 depicts the IS CASSCF natural orbitals common to both PCET pathways, while Secs. S7(A) and S7(B) show the TS and FS CASSCF natural orbitals along the *CHO and *COH paths in Figs. S12-S15.

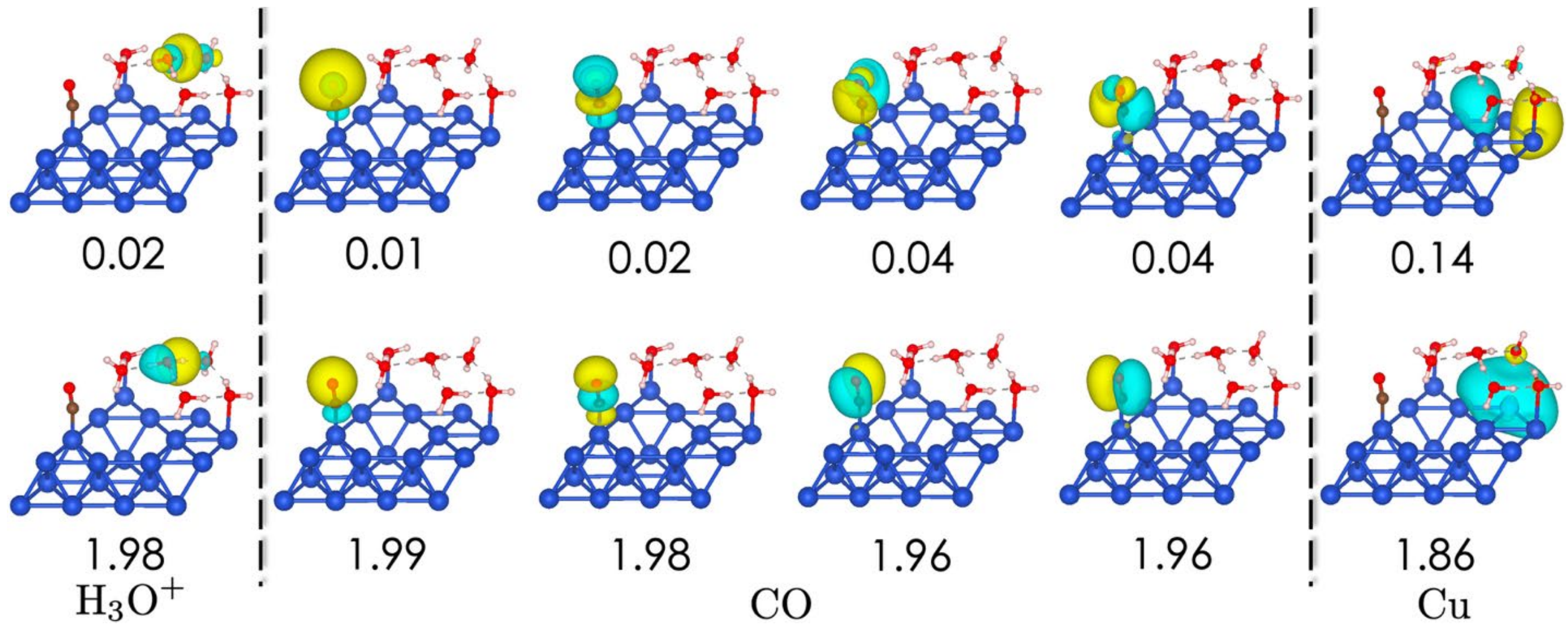

Fig. S11: IS comprising *CO with an Eigen cation and two spectator water molecules on an emb-Cu_{19} cluster: *CO+$H_9O_4^+$+2(H_2O)·emb-Cu_{19}. Natural orbitals from a (12e,12o) AS in emb-CASSCF shown at an isosurface level of ±0.03 a.u. with occupation numbers specified below each orbital; the corresponding canonical orbitals were used as input for emb-MRPT2 calculations. After CASSCF optimization, the orbital character within the (12e,12o) CAS is distributed as (2e,2o) from H_3O^+, (8e,8o) from CO, and (2e,2o) derived from Cu-4*s* orbitals. This IS is the common starting point of both PCET pathways A and B below.

A. *CO → *CHO via PCET

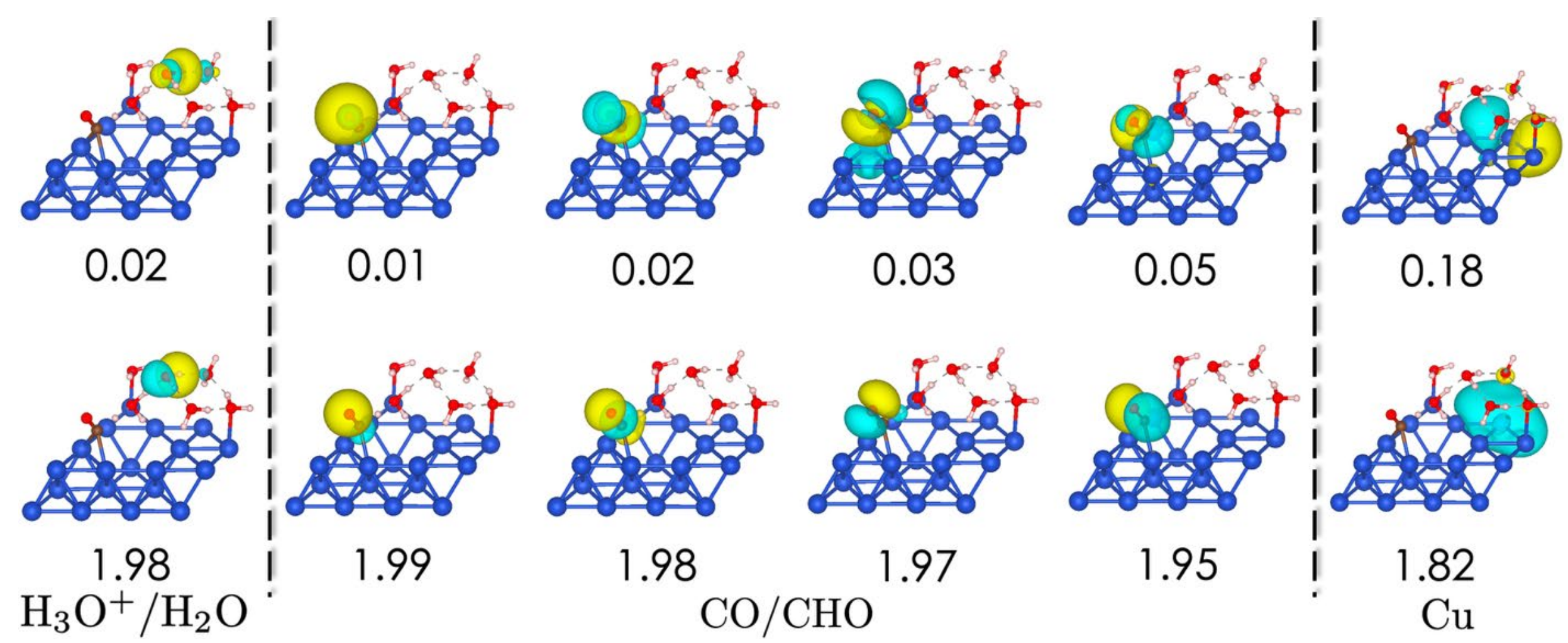

Fig. S12: TS for *CO reacting with an Eigen cation, in the presence of two spectator adsorbed water molecules on an emb-Cu_{19} cluster: *CO–$H_9O_4^+$+2(H_2O)·emb-Cu_{19}. Natural orbitals from a (12e,12o) AS in emb-CASSCF shown at an isosurface level of ±0.03 a.u. with occupation numbers specified below each orbital; the corresponding canonical orbitals were used as input for emb-MRPT2 calculations. After CASSCF optimization, the orbital character within the (12e,12o) CAS is distributed as (2e,2o) from H_3O^+/H_2O, (8e,8o) from CO/CHO, and (2e,2o) derived from Cu-4*s* orbitals.

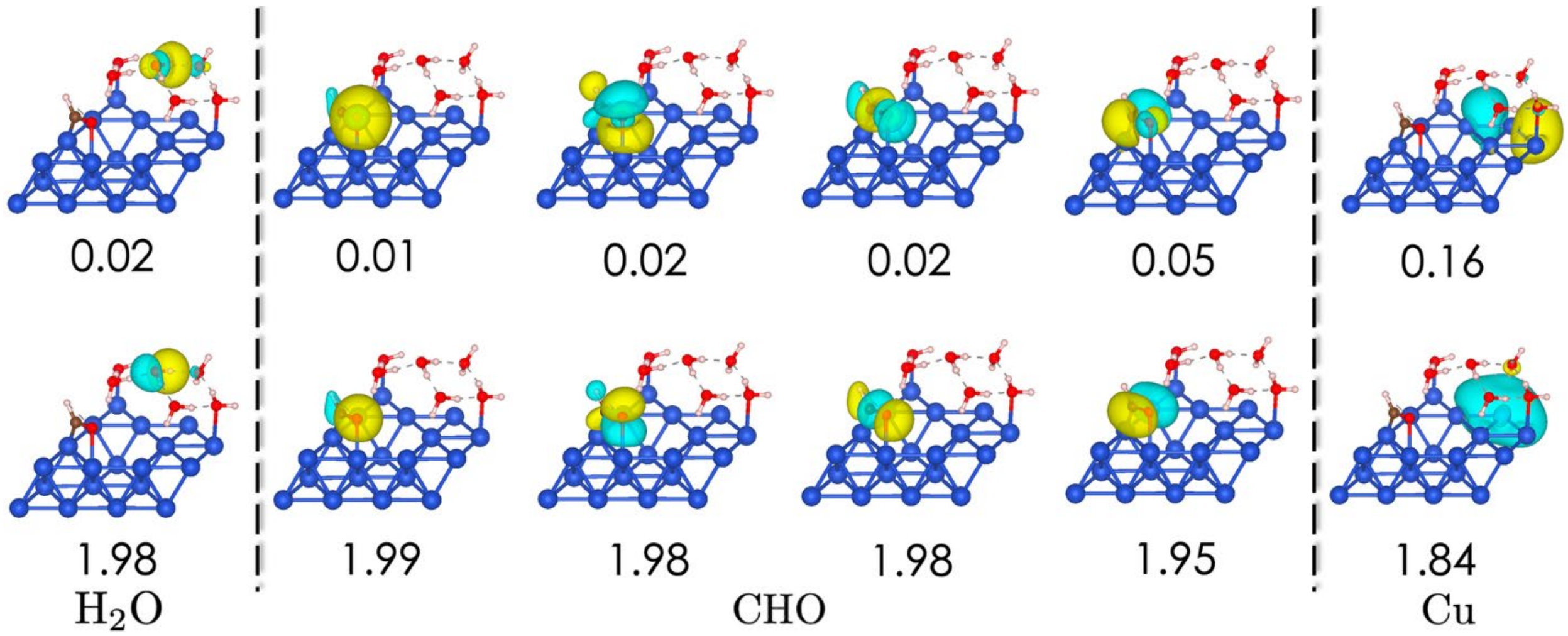

Fig. S13: FS comprising *CHO with four water molecules and two additional spectator adsorbed water molecules on an emb-Cu_{19} cluster: *CHO–$(H_2O)_4$+2(H_2O)·emb-Cu_{19}. Natural orbitals from a (12e,12o) AS in emb-CASSCF shown at an isosurface level of ±0.03 a.u. with occupation numbers specified below each orbital; the corresponding canonical orbitals were used as input for emb-MRPT2 calculations. After CASSCF optimization, the orbital character within the (12e,12o) CAS is distributed as (2e,2o) from H_2O, (8e,8o) from CHO, and (2e,2o) derived from Cu-4*s* orbitals.

B. *CO → *COH via PCET

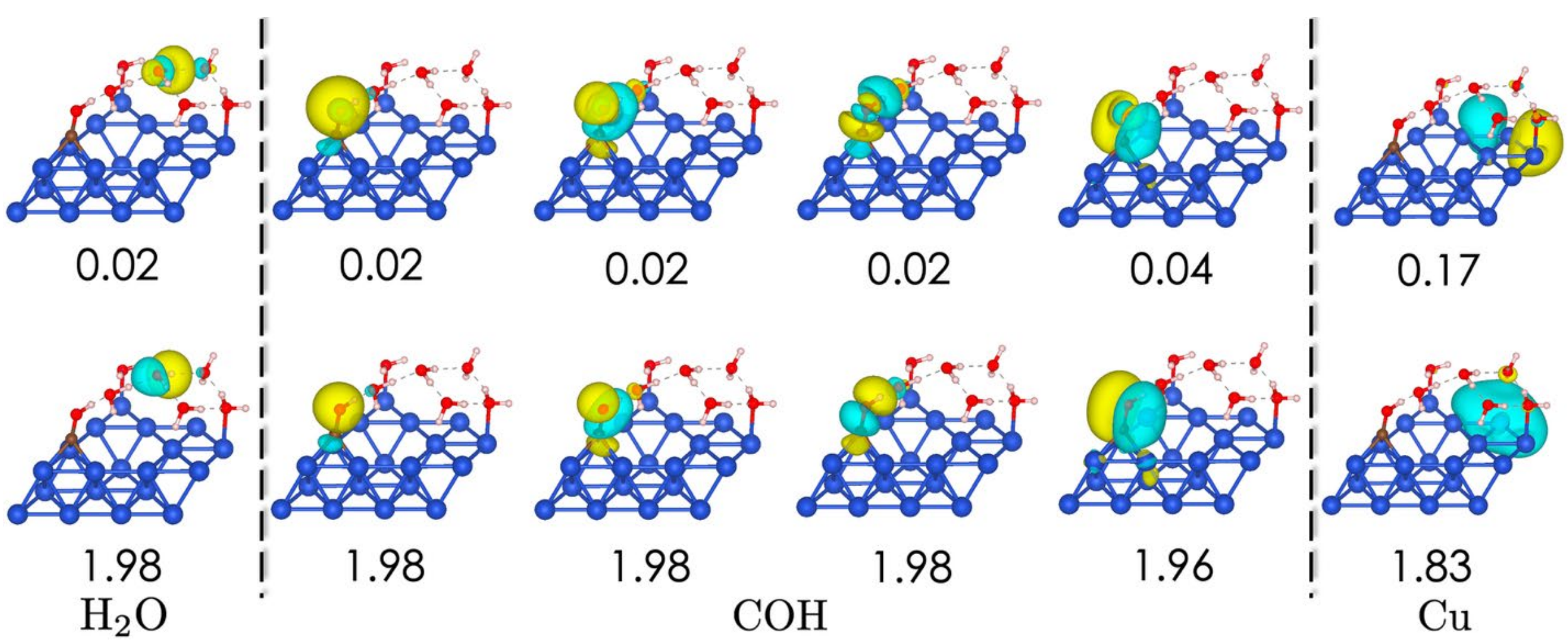

Fig. S14: TS comprising the incipient *COH product with four water molecules and two additional spectator adsorbed water molecules on an emb-Cu_{19} cluster: *COH–$(H_2O)_4$+2(H_2O)·emb-Cu_{19}. Natural orbitals from a (12e,12o) AS in emb-CASSCF shown at an isosurface level of ±0.03 a.u. with occupation numbers specified below each orbital; the corresponding canonical orbitals were used as input for emb-MRPT2 calculations. After CASSCF optimization, the orbital character within the (12e,12o) CAS is distributed as (2e,2o) from H_2O, (8e,8o) from COH, and (2e,2o) derived from Cu-4*s* orbitals.

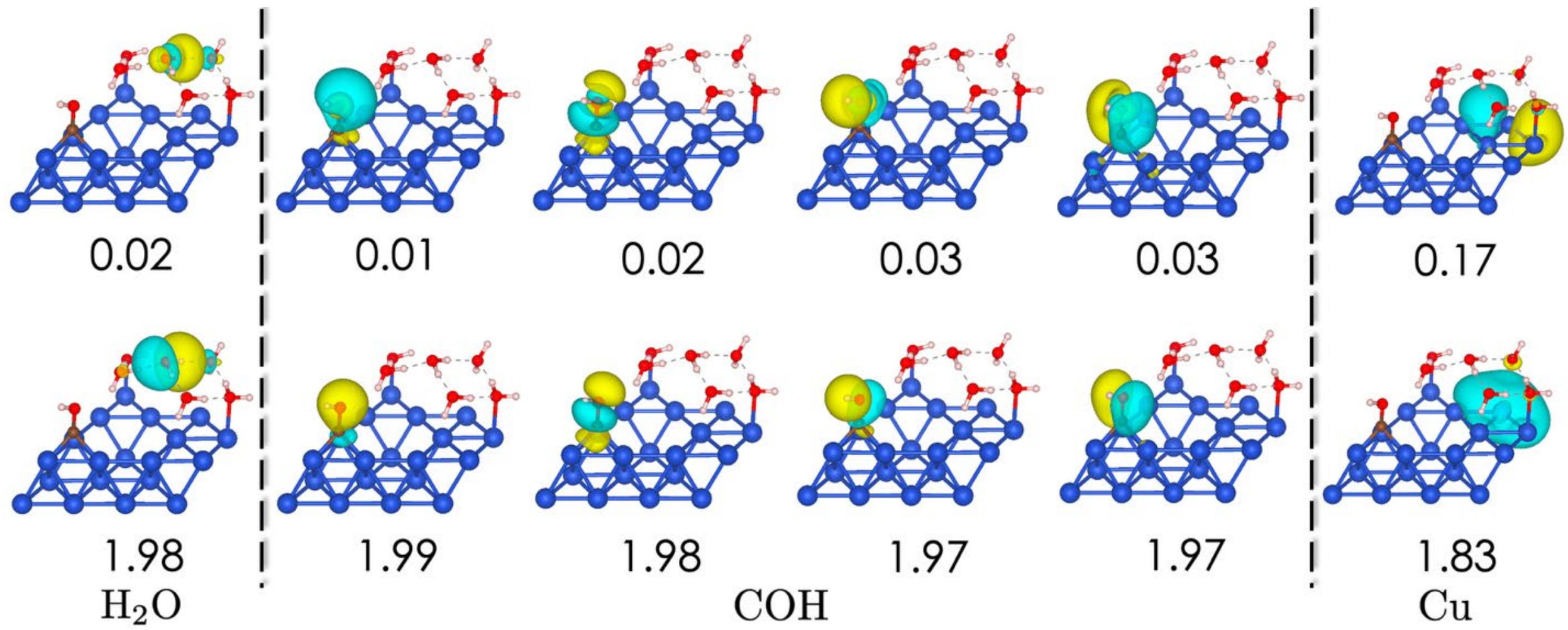

Fig. S15: FS comprising *COH with four water molecules and two additional spectator adsorbed water molecules on an emb-Cu_{19} cluster: *COH–$(H_2O)_4$+2(H_2O)·emb-Cu_{19}. Natural orbitals from a (12e,12o) AS in emb-CASSCF shown at an isosurface level of ±0.03 a.u. with occupation numbers specified below each orbital; the corresponding canonical orbitals were used as input for emb-MRPT2 calculations. After CASSCF optimization, the orbital character within the (12e,12o) CAS is distributed as (2e,2o) from H_2O, (8e,8o) from COH, and (2e,2o) derived from Cu-4*s* orbitals.

S8. Transformation from constant charge to constant potential free energies

Table S2. Constant-charge reaction (ΔE_0^{rxn}) and activation energies ($\Delta E_0^{\ddagger}$), without ZPE or thermal corrections, for the PCET reduction of *CO to *CHO and *COH computed using the [2S]-sol model in PW-DFT, followed by ECW refinements with emb-CASPT2 and emb-NEVPT2.

[2S]-sol	ΔE_0^{rxn} (eV)				$\Delta E_0^{\ddagger}$ (eV)			
**Reaction**	**DFT-PBE-D3(BJ)**	**emb-CASPT2** (12e,12o)	**emb-CASPT2** (14e,14o)	**emb-NEVPT2** (12e,12o)	**DFT-PBE-D3(BJ)**	**emb-CASPT2** (12e,12o)	**emb-CASPT2** (14e,14o)	**emb-NEVPT2** (12e,12o)
*CO + H^+ + e^- → *CHO	0.19	0.10	0.11	0.32	0.78	1.45	1.42	1.74
*CO + H^+ + e^- → *COH	0.45	0.78	0.80	1.10	0.55	1.22	1.24	1.48

Table S3. Constant-charge reaction (ΔG^{rxn}) and activation ($\Delta G^{\ddagger}$) free energies, including ZPE and thermal corrections at T = 298.15 K obtained using [2S]-sol model in PW-DFT and refined with ECW via emb-CASPT2 and emb-NEVPT2.

[2S]-sol	ΔG^{rxn} (eV) [T = 298.15 K, constant # electrons]				$\Delta G^{\ddagger}$ (eV) [T = 298.15 K, constant # electrons]			
**Reaction**	**DFT-PBE-D3(BJ)**	**emb-CASPT2** (12e,12o)	**emb-CASPT2** (14e,14o)	**emb-NEVPT2** (12e,12o)	**DFT-PBE-D3(BJ)**	**emb-CASPT2** (12e,12o)	**emb-CASPT2** (14e,14o)	**emb-NEVPT2** (12e,12o)
*CO + H^+ + e^- → *CHO	0.18	0.09	0.10	0.31	0.64	1.30	1.27	1.60
*CO + H^+ + e^- → *COH	0.47	0.80	0.82	1.12	0.56	1.24	1.26	1.50

In this section, we outline the step-by-step procedure used to determine the parameters entering Eqs. (3) - (6) in the main text. This approach was employed recently for C-C coupling on pristine Cu(100) and is described in detail in the supporting information (SI) of ref. 7.

A. Determination of Δn_e from Bader charge partitioning[8, 9] within the [2S]-sol model

For the reaction $^*CO + H^+ + e^- \rightarrow {}^*CHO$ **[R1]**, $n_e(^*CO)$ and $n_e(^*CHO)$ denote the Bader charges associated with the adsorbed CO and CHO species, respectively. Accordingly,

$$\Delta n_e[a \rightarrow b] = \frac{n_e(2^*CHO) - n_e(2^*CO)}{2}$$

$$\Delta n_e[a \rightarrow b_{TS}] = \frac{n_e(2^*CO\ \text{at TS}) - n_e(2^*CO)}{2}$$

The factors of $\frac{1}{2}$ and 2 (in the numerator) account for the presence of adsorbates on both sides of the slab. At the **[R1]** TS, the proton has yet to fully transfer to *CO.

Similarly, for the reaction $^*CO + H^+ + e^- \rightarrow {}^*COH$ **[R2]**, $n_e(^*CO)$ and $n_e(^*COH)$ denote the Bader charges associated with the adsorbed CO and COH species, respectively. Because **[R1]** and **[R2]** share the same IS, they also have the same value of $n_e(^*CO)$.

$$\Delta n_e[a \rightarrow b] = \frac{n_e(2^*COH) - n_e(2^*CO)}{2}$$

$$\Delta n_e[a \rightarrow b_{TS}] = \frac{n_e(2^*COH\ \text{at TS}) - n_e(2^*CO)}{2}$$

Unlike in [R1], at the [R2] TS, the proton has fully transferred to *CO.

An alternative scheme for computing Δn_e based on ref. 10 was used in refs. 11, 12. This scheme estimates the number of electrons transferred in the electroactive region by calculating the net charge of the slab + adsorbates before and after PCET. We will refer to it as $\Delta n_e'$ here and define it as

$$\Delta n_e'[a \rightarrow b] = 1 - \left\{ \frac{\Delta n_e\left(Cu_{148} + 4(H_2O^*)\right)}{2} + \Delta n_e[a \rightarrow b] \right\},$$

which is an expression adapted from SI Sec. 5 of ref. [7] that directly relates $\Delta n_e'$ to Δn_e. The first term is the number of protons transferred at the FS (or TS), which is 1 for a PCET product (also 1 for a late TS). The term $\Delta n_e\left(Cu_{148} + 4(H_2O^*)\right)$ corresponds to change in the number of electrons in the full Cu periodic slab (148 Cu-atoms with a vacancy on each surface) and the adsorbed spectator waters (2 H_2O on each surface). $\Delta n_e'$s for [**R1**] and [**R2**] are reported within parentheses in Table S4. Note that this alternative scheme was used in ref. 11 to determine the potential dependence of the free energetics on the pristine Cu(111), the results of which are reproduced in Fig. 6 of the main text for comparison.

B. Absolute work function Φ, within the [2S]-sol model

Due to the presence of continuum water, the work functions obtained directly in PW-DFT are referenced to the bulk of the implicit solvent instead of vacuum. We define $\delta\phi = \Phi - \phi_{sol}$, where $\delta\phi = \langle V_H + V_{ion}\rangle_{\text{bulk, continuum}} - E_F$, evaluated for the IS, TS, and FS of reactions **[R1]** and **[R2]** as shown in Fig. S5. To obtain Φ, we must compute the work function of continuum water ϕ_{sol} to add to $\delta\phi$. We can compute ϕ_{sol} by using the Computational Hydrogen Electrode (CHE) model ($\Delta G_{a\to b}$(CHE)),[7, 10, 13-15] to compute the reaction free energy obtained at 0 V vs. the standard hydrogen electrode (SHE; pH=0, U=0).

From DFT-PBE-D3(BJ), we calculate the reaction free energetics of the overall reactions **[R1]** and **[R2]** using the CHE model, $\Delta G_{a\to b}$(CHE), where the free energy of $(H^+ + e^-)$ or $G_{(H^+ + e^-)}$ is replaced by the free energy of half a H_2(g), $\frac{1}{2}G_{H_2}$(*vide infra*):

$$H^+ + e^- \leftrightarrow \frac{1}{2}H_2$$

To calculate $\Delta G_{a\to b}$(CHE), we modified the IS structure used for the PCET pathway by removing an H atom from each side of the slab, i.e., a proton from each hydronium ion (there are two in our [2S]-sol model), resulting in *CO + 4(H_2O) + 2(H_2O) on both sides of the slab, and the two excess electrons in the slab (derived originally from the solvated H_3O precursors that autoionized to form the Eigen cations and the negatively charged surface). The FS remained unmodified. Upon structural relaxation within [2S]-sol, we obtain the free energy of the IS $\left(\frac{G_{2*CO}}{2}\right)$. Therefore, $G_a(\text{CHE}) = \frac{G_{2*CO}}{2} + \frac{1}{2}G_{H_2}$, and $G_b(\text{CHE}) = G_b(\Phi_b)$, as defined above. Finally, $\Delta G_{a\to b}(\text{CHE}) = G_b(\text{CHE}) - G_a(\text{CHE})$.

<u>Free energy of H_2 gas computation</u>: Using the DFT-PBE-D3(BJ) electronic energy,

$$E_0(H_2) = -6.771 \text{ eV},$$

and the ZPE obtained from vibrational analysis of the H–H stretch,

$$\text{ZPE}(H_2) = 0.269 \text{ eV},$$

the free energy of gaseous H_2 at $T = 298.15$ K is computed in eV as

$$G(H_2) = E_0 + \text{ZPE} + \int_0^T C_p(T')\,dT' - TS$$

$$= -6.771 + 0.269 + 0.0878 - 0.404$$

$$= -6.8182 \text{ eV},$$

where the thermal enthalpic (integrated heat capacity at constant pressure C_p) and entropic corrections (TS) were taken from the NIST–JANAF tables.[16]

Finally, without loss of generality, we replace $\Phi \rightarrow \Phi - \phi_{sol}$ in Eqs. (3) and (5) of the main text. Solving for the $\Phi - \phi_{sol}$ at which $\Delta G_{a\rightarrow b}(\Phi - \phi_{sol}) = \Delta G_{a\rightarrow b}(\text{CHE})$ yields Φ at 0 V vs. U_{SHE}:

$$\Phi - \phi_{sol} = \frac{\Delta G_{a\rightarrow b}(\text{CHE}) - \Delta G_{a\rightarrow b}(\Phi - \phi_{sol} = 0)}{\frac{\Delta n_e[a \rightarrow b]}{e}}\ , \tag{S1}$$

where analogous to Eq. (5) of the main text,

$$\Delta G_{a\rightarrow b}(\Phi - \phi_{sol} = 0) = [G_b(\Phi_b - \phi_{sol}) - G_a(\Phi_a - \phi_{sol})]\ - \Delta n_e[a \rightarrow b]\left(\frac{\Phi_b - \phi_{sol} + \Phi_a - \phi_{sol}}{2e}\right) \tag{S2}$$

Note that the rhs of Eq. (S2) was calculated directly using the PCET models. $\Delta G_{a\rightarrow b}(\text{CHE, pH} = 0)$ corresponds to the free energy at 0 V vs. U_{SHE}, therefore at $\Delta G_{a\rightarrow b}(\Phi - \phi_{sol}) = \Delta G_{a\rightarrow b}(\text{CHE at pH = 0})$, Eq. (S1) above must be

$$\Phi - \phi_{sol} = eU_{SHE} - \phi_{sol}\ , \tag{S3}$$

where we set $U_{SHE} = 4.281$ V, the most accurate available experimental value.[17]

Using the values of $\delta\phi$ and Δn_e listed in Table S4 for **[R1]** and **[R2]** and respectively derived from Fig. S5 and Bader charges for the adsorbates, together with the DFT-PBE-D3(BJ) reaction free energies ΔG^{rxn} given in Table S3, we evaluate Eq. (S2) first and then Eq. (S1) with the CHE free energies in Table S5, to finally obtain ϕ_{sol} by using Eq. (S3) with the lhs set equal to the value of Eq. (S1) and rhs defined by the empirical value of U_{SHE} given above, to define Φ; the resulting values are reported in Table S5.

Using $\delta\phi$s and ϕ_{sol}, we calculate the absolute work functions Φ for the IS, TS, and FS of **[R1]** and **[R2]**, which are listed in Table S4. Finally, to reference the absolute work functions Φ to the RHE scale at pH = 0, we apply a constant shift of $U_{RHE} = 4.281$ V, such that $\frac{\Phi}{e} = 4.281$ V corresponds to $\frac{\Phi}{e}$ vs. RHE $= 0$, in other words: $\frac{\Phi}{e}$ vs. RHE (pH = 0) = $\frac{\Phi}{e} - 4.281$ V. ΔG^{rxn} and $\Delta G^{\ddagger}$ for $\frac{\Phi}{e} - U_{RHE} = 0$ V and -0.9 V are listed in Table S6.

Table S4. PW-DFT-PBE vacancy-containing Cu(111) work function $\delta\phi$ in continuum water; number of electrons n_e associated with the eCOR intermediates (corresponding to adsorbate species, e.g., *CO, *CHO, *COH, on both sides of the slab, normalized by a factor of $\frac{1}{2}$)[7] computed from Bader charge partitioning (Sec. S8(A)),[8, 9] and change in the number of electrons Δn_e between the IS and TS, and IS and FS of *CO → *CHO and *CO → *COH pathways. Φ is the absolute work function of the IS, TS and FS for the two PCET eCOR pathways.

*Cu(111)-vacancy	$\delta\phi$ (eV)[a]	n_e	Δn_e	Φ^b(eV)
[IS] $CO+H_9O_4^{+}+2(H_2O)$	3.60	10.34	--	3.61
[TS] $CHO–(H_2O)_4+2(H_2O)$	4.03	10.54	0.20 (0.40)[c]	4.04
[FS] $CHO–(H_2O)_4+2(H_2O)$	4.18	11.40	1.06 (0.83)[c]	4.19
[IS] $CO+H_9O_4^{+}+2(H_2O)$	3.60	10.34	--	3.65
[TS] $COH–(H_2O)_4+2(H_2O)$	4.09	11.27	0.93 (0.80)[c]	4.14
[FS] $COH–(H_2O)_4+2(H_2O)$	4.03	11.30	0.96 (0.81)[c]	4.08

[a] $\delta\phi = \Phi - \phi_{sol} = \langle V_H + V_{ion}\rangle_{bulk,\ continuum} - E_F$ (Sec. S8 text above).
[b] $\Phi = \delta\phi + \phi_{sol}$; ϕ_{sol} taken from Table S5.
[c] Using an alternative expression (Sec. S8A) of the scheme from refs. 10 and 11.

Table S5. DFT-PBE-D3(BJ) reaction free energies with the CHE model (ΔG^{rxn}_{CHE}) in continuum water at $T = 298.15$ K; see text above. Values for Eqs. (S2) and (S1) computed as described in the text above. ϕ_{sol} is the absolute work function of the implicit solvent (in vacuum) computed using the CHE.[7, 10, 11, 13-15]

[CHE model] Reaction	ΔG^{rxn}_{CHE} (eV)[a]	Eq. (S2) (eV)	Eq. (S1) (eV)	ϕ_{sol} (eV)[b]
$*CO + {}^{1}/_{2}\, H_2 \rightarrow *CHO$	0.58	-3.94	4.27	0.01
$*CO + {}^{1}/_{2}\, H_2 \rightarrow *COH$	0.87	-3.18	4.23	0.05

[a] $\Delta G^{rxn}_{CHE} = G_{FS} - G_{CO} - G_{H}$,
[b] follows from Eq. (S1) ≡ Eq. (S3).

Table S6. Potential-dependent reaction (ΔG^{rxn}) and activation ($\Delta G^{\ddagger}$) free energies computed using the Chan-Nørskov capacitor model with the CHE (Sec. S8, Table S5),[7, 10, 11, 13-15] reported at select values of the applied potentials referenced to RHE at pH = 0 (see Fig. 4 in the main text) at DFT-PBE-D3(BJ), emb-CASPT2(12e,12o), emb-CASPT2(14e,14o), and emb-NEVPT2(12e,12o) levels of theory. ΔG^{rxn} and $\Delta G^{\ddagger}$ are computed as linear functions of $\frac{\Phi}{e} - U_{RHE}$ using Eqs. (3) – (6) in the main text. $U_{RHE} = 4.281$ V at pH = 0 is the reversible hydrogen electrode potential.[17]

**DFT-PBE-D3(BJ)**		**ΔG^{rxn} (eV)**			**$\Delta G^{\ddagger}$ (eV)**	
**Reaction**	**Δn_e [IS, FS]**	$\frac{\Phi}{e} - U_{RHE} = 0$ V	$\frac{\Phi}{e} - U_{RHE} = -0.9$ V	**Δn_e [IS, TS]**	$\frac{\Phi}{e} - U_{RHE} = 0$ V	$\frac{\Phi}{e} - U_{RHE} = -0.9$ V
$*CO + H^+ + e^- \rightarrow *CHO$	1.06	0.58	-0.37	0.20	0.73	0.55
$*CO + H^+ + e^- \rightarrow *COH$	0.96	0.88	0.01	0.93	0.93	0.09
**emb-CASPT2(12e,12o)**		**ΔG^{rxn} (eV)**				**$\Delta G^{\ddagger}$ (eV)**
**Reaction**	**Δn_e [IS, FS]**	$\frac{\Phi}{e} - U_{RHE} = 0$ V	$\frac{\Phi}{e} - U_{RHE} = -0.9$ V	**Δn_e [IS, TS]**	$\frac{\Phi}{e} - U_{RHE} = 0$ V	$\frac{\Phi}{e} - U_{RHE} = -0.9$ V
$*CO + H^+ + e^- \rightarrow *CHO$	1.06	0.49	-0.46	0.20	1.40	1.22
$*CO + H^+ + e^- \rightarrow *COH$	0.96	1.20	0.34	0.93	1.60	0.76
**emb-CASPT2(14e,14o)**		**ΔG^{rxn} (eV)**				**$\Delta G^{\ddagger}$ (eV)**
**Reaction**	**Δn_e [IS, FS]**	$\frac{\Phi}{e} - U_{RHE} = 0$ V	$\frac{\Phi}{e} - U_{RHE} = -0.9$ V	**Δn_e [IS, TS]**	$\frac{\Phi}{e} - U_{RHE} = 0$ V	$\frac{\Phi}{e} - U_{RHE} = -0.9$ V
$*CO + H^+ + e^- \rightarrow *CHO$	1.06	0.50	-0.46	0.20	1.36	1.18
$*CO + H^+ + e^- \rightarrow *COH$	0.96	1.22	0.36	0.93	1.62	0.78
**emb-NEVPT2(12e,12o)**		**ΔG^{rxn} (eV)**				**$\Delta G^{\ddagger}$ (eV)**
**Reaction**	**Δn_e [IS, FS]**	$\frac{\Phi}{e} - U_{RHE} = 0$ V	$\frac{\Phi}{e} - U_{RHE} = -0.9$ V	**Δn_e [IS, TS]**	$\frac{\Phi}{e} - U_{RHE} = 0$ V	$\frac{\Phi}{e} - U_{RHE} = -0.9$ V
$*CO + H^+ + e^- \rightarrow *CHO$	1.06	0.72	-0.24	0.20	1.69	1.51
$*CO + H^+ + e^- \rightarrow *COH$	0.96	1.52	0.66	0.93	1.86	1.02

Supplementary References

(1) Mathew, K.; Kolluru, V. S. C.; Mula, S.; Steinmann, S. N.; Hennig, R. G. Implicit self-consistent electrolyte model in plane-wave density-functional theory. *The Journal of Chemical Physics* **2019**, *151* (23), 234101. DOI: 10.1063/1.5132354.

(2) Gauthier, J. A.; Ringe, S.; Dickens, C. F.; Garza, A. J.; Bell, A. T.; Head-Gordon, M.; Nørskov, J. K.; Chan, K. Challenges in Modeling Electrochemical Reaction Energetics with Polarizable Continuum Models. *ACS Catalysis* **2019**, *9* (2), 920-931. DOI: 10.1021/acscatal.8b02793.

(3) Huang, C.; Pavone, M.; Carter, E. A. Quantum mechanical embedding theory based on a unique embedding potential. *The Journal of Chemical Physics* **2011**, *134* (15), 154110. DOI: 10.1063/1.3577516.

(4) Libisch, F.; Huang, C.; Carter, E. A. Embedded Correlated Wavefunction Schemes: Theory and Applications. *Accounts of Chemical Research* **2014**, *47* (9), 2768-2775. DOI: 10.1021/ar500086h.

(5) Yu, K.; Libisch, F.; Carter, E. A. Implementation of density functional embedding theory within the projector-augmented-wave method and applications to semiconductor defect states. *The Journal of Chemical Physics* **2015**, *143* (10), 102806. DOI: 10.1063/1.4922260.
(6) Yu, K.; Krauter, C. M.; Dieterich, J. M.; Carter, E. A. Density and Potential Functional Embedding: Theory and Practice. In *Fragmentation: Toward Accurate Calculations on Complex Molecular Systems*, 2017; pp 81-117, Mark Gordon, Ed. (John Wiley & Sons), ISBN: 978-111-119-12924-12920.
(7) Martirez, J. M. P.; Carter, E. A. C–C Bond Formation during Electrochemical CO2 Reduction on Pristine Cu(100) Unlikely to Involve Adsorbed CO at Any Potential. *Journal of the American Chemical Society* **2026**, *148* (7), 7415-7425. DOI: 10.1021/jacs.5c20300.
(8) Tang, W.; Sanville, E.; Henkelman, G. A grid-based Bader analysis algorithm without lattice bias. *Journal of Physics: Condensed Matter* **2009**, *21* (8), 084204. DOI: 10.1088/0953-8984/21/8/084204.
(9) Yu, M.; Trinkle, D. R. Accurate and efficient algorithm for Bader charge integration. *The Journal of Chemical Physics* **2011**, *134* (6), 064111. DOI: 10.1063/1.3553716.
(10) Chan, K.; Nørskov, J. K. Electrochemical Barriers Made Simple. *The Journal of Physical Chemistry Letters* **2015**, *6* (14), 2663-2668. DOI: 10.1021/acs.jpclett.5b01043.
(11) Zhao, Q.; Martirez, J. M. P.; Carter, E. A. Revisiting Understanding of Electrochemical CO2 Reduction on Cu(111): Competing Proton-Coupled Electron Transfer Reaction Mechanisms Revealed by Embedded Correlated Wavefunction Theory. *Journal of the American Chemical Society* **2021**, *143* (16), 6152-6164. DOI: 10.1021/jacs.1c00880.
(12) Zhao, Q.; Martirez, J. M. P.; Carter, E. A. Electrochemical Hydrogenation of CO on Cu(100): Insights from Accurate Multiconfigurational Wavefunction Methods. *The Journal of Physical Chemistry Letters* **2022**, *13* (44), 10282-10290. DOI: 10.1021/acs.jpclett.2c02444.
(13) Nørskov, J. K.; Rossmeisl, J.; Logadottir, A.; Lindqvist, L.; Kitchin, J. R.; Bligaard, T.; Jónsson, H. Origin of the Overpotential for Oxygen Reduction at a Fuel-Cell Cathode. *The Journal of Physical Chemistry B* **2004**, *108* (46), 17886-17892. DOI: 10.1021/jp047349j.
(14) Chan, K.; Nørskov, J. K. Potential Dependence of Electrochemical Barriers from ab Initio Calculations. *The Journal of Physical Chemistry Letters* **2016**, *7* (9), 1686-1690. DOI: 10.1021/acs.jpclett.6b00382.
(15) Li, L.; Martirez, J. M. P.; Carter, E. A. Prediction of Highly Selective Electrocatalytic Nitrogen Reduction at Low Overpotential on a Mo-Doped g-GaN Monolayer. *ACS Catalysis* **2020**, *10* (21), 12841-12857. DOI: 10.1021/acscatal.0c03140.
(16) Chase, M. W.; Davies, C. A.; Downey, J. R.; Frurip, D. J.; McDonald, R. A.; Syverud, A. N. *NIST JANAF Thermochemical Tables 1985*; National Institute of Standards and Technology, 1986.
(17) Isse, A. A.; Gennaro, A. Absolute Potential of the Standard Hydrogen Electrode and the Problem of Interconversion of Potentials in Different Solvents. *The Journal of Physical Chemistry B* **2010**, *114* (23), 7894-7899. DOI: 10.1021/jp100402x.
